# Multiple myosin motors interact with sodium/potassium-ATPase alpha 1 subunits

**DOI:** 10.1186/s13041-018-0388-1

**Published:** 2018-08-07

**Authors:** Bhagirathi Dash, Sulayman D. Dib-Hajj, Stephen G. Waxman

**Affiliations:** 1 Department of Neurology, Yale University Schoolof Medicine, New Haven, CT 06510 USA; 2 Center for Neuroscience & Regeneration Research, Yale University School of Medicine, New Haven, CT 06510 USA; 3 Rehabilitation Research center, VA Connecticut Healthcare System, 950 Campbell Avenue, Bldg. 34, West Haven, CT 06516 USA

**Keywords:** Myosins, Class II non-muscle myosins, myh9, myh10, myh14, Myosin Va, Myosin VI, Sodium/potassium ATPase, Sodium pump, Na^+^/K^+^-ATPase α1 subunits

## Abstract

The alpha1 (α1) subunit of the sodium/potassium ATPase (i.e., Na^+^/K^+^-ATPase α1), the prototypical sodium pump, is expressed in each eukaryotic cell. They pump out three sodium ions in exchange for two extracellular potassium ions to establish a cellular electrochemical gradient important for firing of neuronal and cardiac action potentials. We hypothesized that myosin (myo or myh) motor proteins might interact with Na^+^/K^+^-ATPase α1 subunits in order for them to play an important role in the transport and trafficking of sodium pump. To this end immunoassays were performed to determine whether class II non-muscle myosins (i.e., NMHC-IIA/myh9, NMHC-IIB/myh10 or NMHC-IIC/myh14), myosin Va (myoVa) and myosin VI (myoVI) would interact with Na^+^/K^+^-ATPase α1 subunits. Immunoprecipitation of myh9, myh10, myh14, myoVa and myoVI from rat brain tissues led to the co-immunoprecipitation of Na^+^/K^+^-ATPase α1 subunits expressed there. Heterologous expression studies using HEK293 cells indicated that recombinant myh9, myh10, myh14 and myoVI interact with Na^+^/K^+^-ATPase α1 subunits expressed in HEK293 cells. Additional results indicated that loss of tail regions in recombinant myh9, myh10, myh14 and myoVI did not affect their interaction with Na^+^/K^+^-ATPase α1 subunits. However, recombinant myh9, myh10 and myh14 mutants having reduced or no actin binding ability, as a result of loss of their actin binding sites, displayed greatly reduced or null interaction with Na^+^/K^+^-ATPase α1 subunits. These results suggested the involvement of the actin binding site, but not tail regions, of NMHC-IIs in their interaction with Na^+^/K^+^-ATPase α1 subunits. Overall these results suggest a role for these diverse myosins in the trafficking and transport of sodium pump in neuronal and non-neuronal tissues.

**Electronic supplementary material:**

The online version of this article (10.1186/s13041-018-0388-1) contains supplementary material, which is available to authorized users.

## Introduction

The sodium pump (sodium/potassium ATPase; Na^+^/K^+^-ATPase) is an integral membrane protein found in the cells of all higher eukaryotes [1]. It utilizes ATP as a driving force to pump out three sodium ions in exchange for two extracellular potassium ions which establishes both a chemical and an electrical gradient across the cell membrane. The electrical gradient is essential for maintaining cellular resting potential and excitability of myocytes (skeletal and cardiac) and neurons. The sodium gradient helps drive various transport processes such as the fluid reabsorption and translocation of glucose, amino acids, and other nutrients into the cells [1, 2]. Sodium/potassium-ATPase also function as a signaling molecule and is shown to regulate MAPK pathway, reactive oxygen species (ROS) formation as well as intracellular calcium [2–4]. It is principally composed of two subunits, an alpha-subunit (α; ≈100 kDa) and a beta-subunit (β; ≈40 kDa). The α subunit (i.e., catalytic subunit) has four isoforms: α1 (*Atp1a1*), α2 (*Atp1a2*), α3 (*Atp1a3*) and α4 (*Atp1a4*). The β subunits has four isoforms as well: β1 (*Atp1b1*), β2 (*Atp1b2*), β3 (*Atp1b3*) and β4 (*Atp1b4*). These α and β subunits admix to form a minimal functional sodium pump. The minimal functional unit of the Na^+^/K^+^-ATPase can be further modified by a third FXYD subunit (also known as gamma-subunit (γ)) [5]. Mammals express seven FXYD proteins. Na^+^/K^+^-ATPase α1 subunits in association with its β1 subunit is found in nearly every tissue [5] . The α2 subunits are predominantly expressed in adipocytes, muscle, heart, and brain (i.e., mostly glial cells). The α3 subunits are abundant in nervous tissues (i.e., mostly neurons). The α4 subunits are a testis-specific isoform [6]. Ablation of Na^+^/K^+^-ATPase α1, α2 or α3 subunits result in the death of the animal [7].

The mechanisms by which Na^+^/K^+^-ATPase α and β subunits are trafficked to the cell surface are not well understood. Generally, vesicles carrying membrane proteins traffic from the intracellular pools to the plasma membranes. This involves their transport by kinesin family of motor proteins (KIF) along the microtubules and/or by myosin (myo or myh) family of motor proteins (myo or myh) along the actin filaments. Kinesin light chain 2 (KLC2) of kinesin-1 heavy chain (KIF5B) has been shown to be involved in the trafficking of Na^+^/K^+^-ATPase α1 subunits in alveolar epithelial cells [8]. In the same cell types, myosin Va (myoVa), a member of the myosin family of actin-based motor proteins, is shown to be involved in the trafficking of Na^+^/K^+^-ATPase α1 subunits [9].

Class II non-muscle myosins (NM-II), like class II muscle myosins, are hexameric molecules comprising of two heavy chains (HC), two myosin essential light chains (ELCs) and two regulatory light chains (RLCs or MRLCs). The non-muscle myosin heavy chain (NMHC) is comprised of a globular head/motor domain (the site for interaction with actin and adenosine triphosphate (ATP)); a neck region (site for interaction with ELCs and RLCs); and a tail region which homodimerizes in a helical fashion (and possibly the site for interaction with the cargo) [10]. By contrast, myoVI is a monomeric heavy chain that consists of a head domain, a neck region (that contains converter/reverse gear domain, IQ motif/domain and site for interaction with calmodulin light chain) and a tail domain that contains a cargo-binding domain (CBD, the site for association with cargo adaptors). MyoVI moves toward the slow-growing (minus) ends of the actin filaments contrary to all other native myosins that move toward the fast-growing (plus) ends of F-actin [11]. We hypothesized that myosins (myh or myo), particularly class II non-muscle myosins (NM-II; myh9, myh10 and myh14) and myosin VI (myoVI) in addition to myoVa, might play an important role in the trafficking of Na^+^/K^+^-ATPase α1 subunits to cell membranes.

## Methods

### Bioinformatics analyses

Mammalian non-muscle myosin II isoforms were aligned using ClustalO program (https://www.ebi.ac.uk/Tools/msa/clustalo/). The 3-D coordinates of human myh14 (PDB: 5I4E) was retrieved from protein data bank [12]. Structural features of myh14 was visualized and analyzed using UCSF Chimera (http://www.cgl.ucsf.edu/chimera/).

### Antibodies

Various polyclonal (rabbit or goat) antibodies and monoclonal (mouse) antibodies used for immunoprecipitation (IP) and/or immunoblotting (IB) are provided in the supporting information section (Additional file 1: Tables S1 and S2). Antibody dilutions and/or concentrations used for IP and/or IB assays along with the molecular weight (~kDa) of the antigens detected by these antibodies are also provided (Additional file 1: Table S2). Immunoblots were incubated with monoclonal antibodies against myh9 (1:500; Abcam), myh10 (1:1000; Abcam), myosin regulatory light chain (MRLC) (1:200; Santa Cruz Biotechnology), Na^+^/K^+^-ATPase α1 (0.5 μg/mL; DSHB), Na^+^/K^+^-ATPase α1 (1:1000; EMD Millipore), Na^+^/K^+^-ATPase α (1:200; Santa Cruz Biotechnology), GFP (1:1000, NeuroMab), myosin VI (1:500; Sigma), voltage gated sodium channel α subunits (pan-Na_v_α; 1:1000; Sigma) and β-actin (1:10000; Sigma). Polyclonal antibodies against GFP (1:2000; Abcam), mCherry (1:1000; Abcam) and myosin Va (1:500; Cell Signaling Technology) were also used for immunoblotting.

Na^+^/K^+^ ATPase α antibody obtained from Santa Cruz Biotechnology (sc-58,628; clone M7-PB-E9) is considered as a pan-Na^+^/K^+^ ATPase α antibody in some quarters as it detects Na^+^/K^+^ ATPase α subunits (i.e., α1, α2 and α3) from human, mouse, sheep, dog, pig and chicken. It also detects rat Na^+^/K^+^ ATPase α3 but not rat Na^+^/K^+^ ATPase α1 or α2 subunits. The monoclonal antibody against Na^+^/K^+^ ATPase α1 (α6F) was developed by Dr. D.M. Fambrough. It was obtained from the Developmental Studies Hybridoma Bank (DSHB) and was developed under the auspices of NIHCD and maintained by the University of Iowa, Department of Biological Sciences, Iowa City, IA 52242.

The MRLC antibody used in this study (sc-28,329; clone E4; SCBT) is already shown to detect various MRLCs and hence considered as a pan-MRLC in some quarters [13]. It is claimed to recognize the MRLCs from human (i.e., MRCL3, MRLC2, MYL9 and LOC391722: myosin regulatory light chain 12B-like), mouse (i.e., Mylc2b, Myl9 and Myl12a) and rat (i.e., Mrlcb and Myl9) tissues. In our hand, it poorly detects the MRLCs from HEK293 cell lysates, but it detects the MRLCs as a co-immunoprecipitate from precipitation of NMHC-IIs very well (Additional file 2: Figure S1).

### Molecular cloning

Standard molecular cloning or fast cloning methods [14] were followed for sub-cloning. Details about the various cDNA constructs (i.e., species, isoform, amino acid length; etc.) used in this study are provided in the supporting information section (Additional file 1: Table S3).

### Myosin constructs

CMV-GFP-NMHC II-A (i.e., GFP-myh9), CMV-GFP-NMHC II-B (i.e., GFP-myh10) and EGFP-NMHC II-C (i.e., myh14-GFP) were gifts from Robert Adelstein (Additional file 1: Table S3) [15, 16]. Like myh14-GFP, myh9 and myh10 were sub-cloned to have GFP fused to their C-termini (i.e., myh9-GFP and myh10-GFP). The tail regions of myh9 (i.e., AAs:1928–1960), myh10 (i.e., AAs:1934–1976) and myh14 (i.e., AAs:1946–1992) were deleted to make myh9-Δatil-GFP, myh10-Δtail-GFP and myh14-Δtail-GFP constructs respectively following the work of others [17]. The actin binding sites (ABS) of myh9 (i.e., AAs:654–676), myh10 (i.e., 661–683) and myh14 (i.e., 674–696) were also deleted to make myh9-ΔABS-GFP, myh9-ΔABS-GFP and myh9-ΔABS-GFP constructs respectively. Information about the actin binding sites and/or tail regions of NMHC-IIs were obtained from literature and/or UniProt site [17–19].

The human myosin Va (myoVa) and myosin VI (myoVI) cDNA clones were obtained from Dharmacon (Lafayette, CO, USA) (Additional file 1: Table S3). Both myoVa and myoVI were sub-cloned to have GFP fused to their C-termini (i.e., myoVa-GFP and myoVI-GFP, respectively). MyoVI was also tagged with mCherry in the N-terminus (i.e., mCherry-MyoVI). Twenty two (22), 60 and 120 AAs were deleted from the C-terminal end of mCherry-MyoVI to make mCherry-Myo6-ΔT1, mCherry-Myo6-ΔT2 and mCherry-Myo6-ΔT3 constructs respectively following the work of others [20].

### Non-myosin constructs

A mouse Na^+^/K^+^-ATPase α1 cDNA clone was obtained from Dharmacon (Lafayette, CO, USA). Na^+^/K^+^-ATPase α1 subunits were sub-cloned to have mCherry fused into their C-terminus (i.e., Na^+^/K^+^-ATPase α1-mCherry). The ankyrin-G-270-mCherery (i.e., AnkG270-mCherry) construct was a gift from Benedicte Dargent [21] (Additional file 1: Table S3). The Na_v_1.6 (i.e., voltage gated sodium channel alpha 6 subunit/SCN8A) construct was available in our laboratory. It harbors a mutation [Tyr371Ser] that renders it resistant to tetrodotoxin (TTX).

### Cell culture and transfection

HEK293 cells were cultured according to standard procedures and were transfected with desired cDNA constructs using Optifect (Thermo Fisher Scientific, Waltham, MA) or LipoJet™ transfection reagent (SignaGen Laboratories, Rockville, MD) according to manufacturer’s instructions.

### Preparation of cell and tissues lysates

All animal care and experimental studies were approved by the Veterans Administration Connecticut Healthcare System Institutional Animal Care and Use Committee. We followed the protocols published elsewhere [22] with some modifications to prepare the adult rat (Sprague-Dawley) brain tissue or HEK293 cell lysates for IP and immunoblotting. The lysis or IP buffer was made of 20 mM Tris-Cl (pH 7.4), 150 mM NaCl, 1% Triton X-100, 1 mM DTT, 10 mM EGTA and 2× Complete protease inhibitor cocktail (Roche Diagnostics Corporation, Indianapolis, IN). An adult whole rat (male or female) brain was homogenized in pieces in a tissue grinder (Qiagen, Valencia, CA) to a final volume ~ 50 mL lysis buffer. Homogenates were solubilized for 2 h at 4 °C, and centrifuged at 50,000 g for 30 min at 4 °C using a Beckman Coulter Optima® ultra-centrifuge to collect the supernatants for immunoprecipitation (IP) and immunoblotting.

Non-transfected HEK293 cells (control) or those transiently transfected with plasmid constructs were collected by centrifugation at 500 g for 5 min at 4 °C upon trypsinization. These pellets were washed twice with ice cold PBS by centrifugation at 500 g for 5 min at 4 °C before lysis using the IP buffer. Cell supernatants were obtained by centrifugation at 15,000 g for 20 min at 4 °C for IP and immunoblotting.

Protein concentration in the tissue lysates was determined using the Bradford reagent (Bio-Rad, Hercules, CA).

### Immunoprecipitation

For IP experiment, HEK293 cell or rat brain tissue supernatants containing 1–4 mg protein (in ~ 1 mL lysate) was pre-cleared (PC) for 1–4 h at 4 °C with 5–10 μg of suitable mouse antibody isotypes, rabbit immunoglobulins or goat immunoglobulins, and 80–100 μl of Dynabead® protein G (Thermo Fisher Scientific). Precleared supernatants were incubated (overnight, 4 °C) with 5–10 μg of desired IP antibody (Additional file 1: Table S1) and 80–100 μl of Dynabead® protein G. Dynabead® Protein G beads bound to control antibody isotypes (i.e., PC complexes) or desired primary antibodies (i.e., IP complexes) were washed for 5 times with IP buffer or wash buffer supplied by the vendor (Thermo Fisher Scientific) and eluted with NuPAGE® LDS Sample Buffer (Thermo Fisher Scientific) in the presence of NuPAGE® Sample Reducing Agent (Thermo Fisher Scientific).

### Western blotting

About 30–50 μg of HEK293 cell or rat brain tissue lysates were denatured using NuPAGE® Sample Reducing Agent in the presence of NuPAGE® LDS Sample Buffer to serve as input (In) sample for western blotting. The input samples, PC complexes, IP complexes and/or at times denatured depleted supernatants (DS) were resolved on NuPAGE® Novex® 4–12% Bis-Tris Gels (1.0 mm, 12 well) and transferred to a nitrocellulose membrane. Membranes were blocked using a blocking buffer (5% non-fat dry milk and 1% BSA in 0.1% TBST or 5% BSA in 0.1% TBST) for 1 h, washed and incubated overnight with desired primary antibodies (Additional file 1: Table S2) diluted in the blocking buffer. The blots were washed and incubated in horseradish peroxidase-conjugated goat anti-mouse (1:10000; Dako, Santa Clara, CA), goat anti-rabbit (1:10000; Dako, Santa Clara, CA) or donkey anti-goat (1:5000) immunoglobulins for 1 h. The blots were washed extensively and developed for 1 to 10 min with the Perkin Elmer Western Lightning Plus-enhanced chemiluminescence (ECL) kit using a Bio-Rad ChemiDoc XRS+ or ChemiDoc Imaging System. At times immunoblots were stripped using a stripping buffer (Thermo Fisher Scientific) to re-probe with another primary antibody.

We usually cut through the IgG-HC and/or IgG-LC regions of the Ponceau S (Sigma) stained nitrocellulose membranes for probing different section of the membrane with different antibodies. Therefore, cut marks could be seen in some images.

## Results

### Antibody characterization

**Fig. 1:**
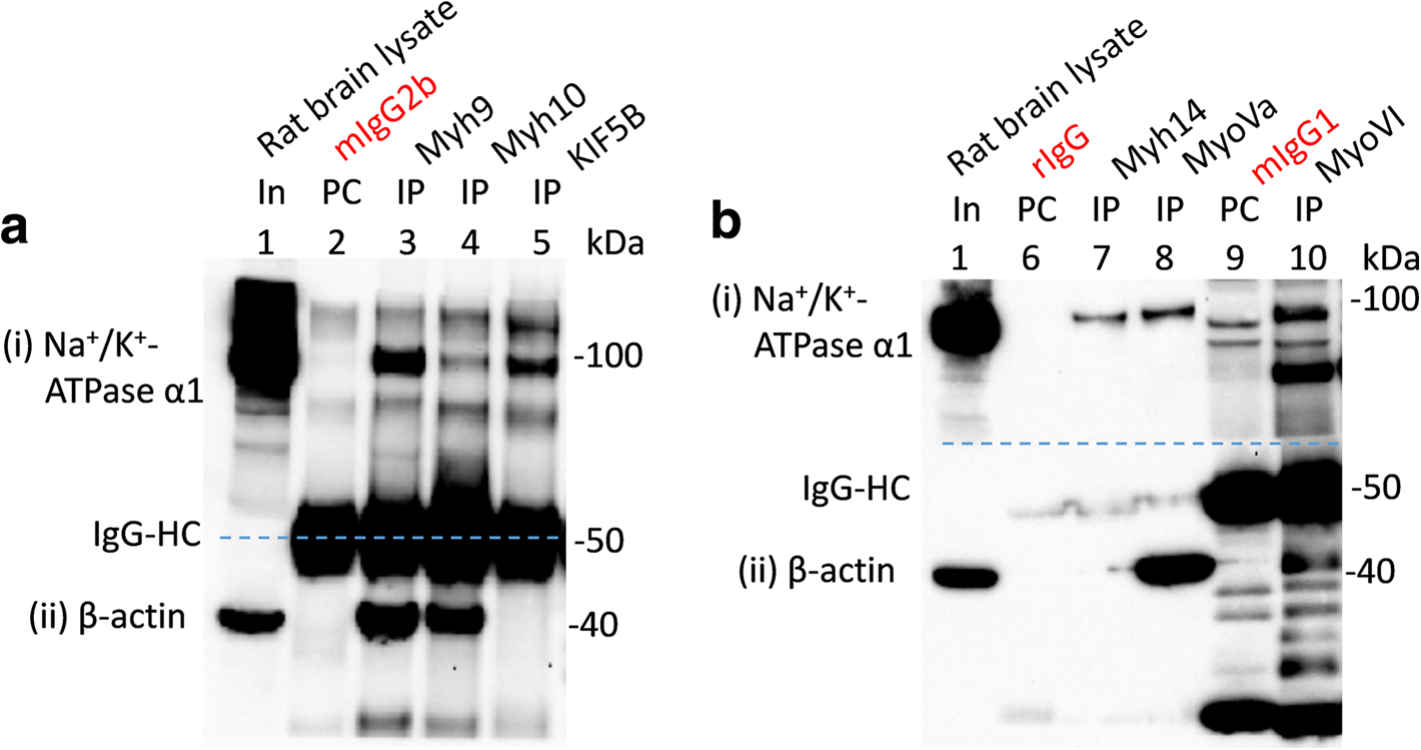
Interaction of multiple myosins with Na^+^/K^+^-ATPase α1 subunits expressed in rat brain. WT adult rat brain lysates (In, lane 1 in **a** and **b**) were precleared (PC) with indicated immunoglobulin isotypes (PC; lane2 = mIgG2b, lane 6 = rIgG and lane 9 = mIgG1) prior to immunoprecipitation (IP) using indicated antibodies (IP; lane 3 = myh9, lane 4 = myh10, lane 5 = KIF5B, lane 7 = Myh14, lane 8 = myoVa and lane 10 = myoVI). Loading of PC complexes in the gel preceded those of the IP complexes. Na^+^/K^+^-ATPase α1 subunits (i) were co-immunoprecipitated with myh9, myh10, KIF5B, myh14, myoVa and myoVI expressed in rat brain tissues. Co-immunoprecipitation of Na^+^/K^+^-ATPase α1 subunits by KIF5B served as a positive control. All the myosins assayed co-immunoprecipitated β-actin (ii). Denatured mouse IgG-HC (i.e., lanes 2–5, 9 and 10; panel (ii)), but not those of rabbit IgG (i.e., lanes 6–8) separated from their intact immunoglobulins (that is used for PC or IP) could be seen as this section was probed with mouse anti-β-actin antibodies

**Fig. 2:**
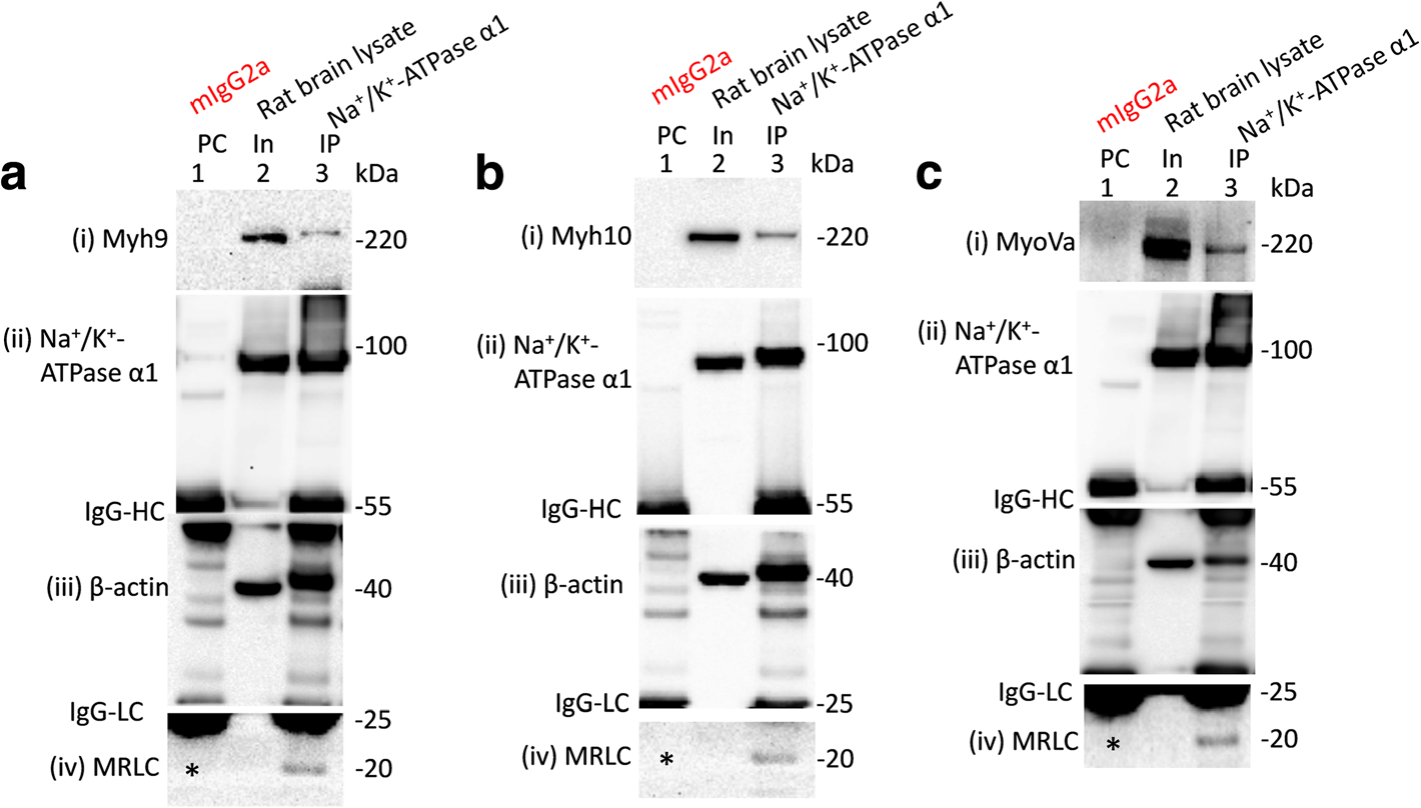
Na^+^/K^+^-ATPase α1 subunits co-immunoprecipitate multiple myosins expressed in adult rat brain. WT adult rat brain lysates (In, lane 2 in **a**, **b** and **c**) were precleared (PC) with mouse IgG1 antibodies (PC, lane 1 in **a**, **b** and **c**) prior to immunoprecipitation (IP) using mouse anti-Na^+^/K^+^-ATPase α1 antibodies of the IgG1 isotypes (IP, lane 3 in **a**, **b** and **c**). Loading of PC complexes in the gel preceded those of the lysate inputs (In). Na^+^/K^+^-ATPase α1 subunits co-immunoprecipitated myh9 (**a** (i)), myh10 (**b**, (i)), myoVa (**c** (i)), β-actin ((iii) in **a**, **b** and **c**) and myosin regulatory light chain (MRLC) ((iv) in **a**, **b** and **c**) from rat brain. An asterisk (‘*’; (iv) in **a**, **b** and **c**) indicates lack of detection of the input signal for the MRLCs. As expected anti-Na^+^/K^+^-ATPase α1 antibodies immunoprecipitated Na^+^/K^+^-ATPase α1 subunits expressed in brain tissues ((ii) in **a**, **b** and **c**)

**Fig. 3:**
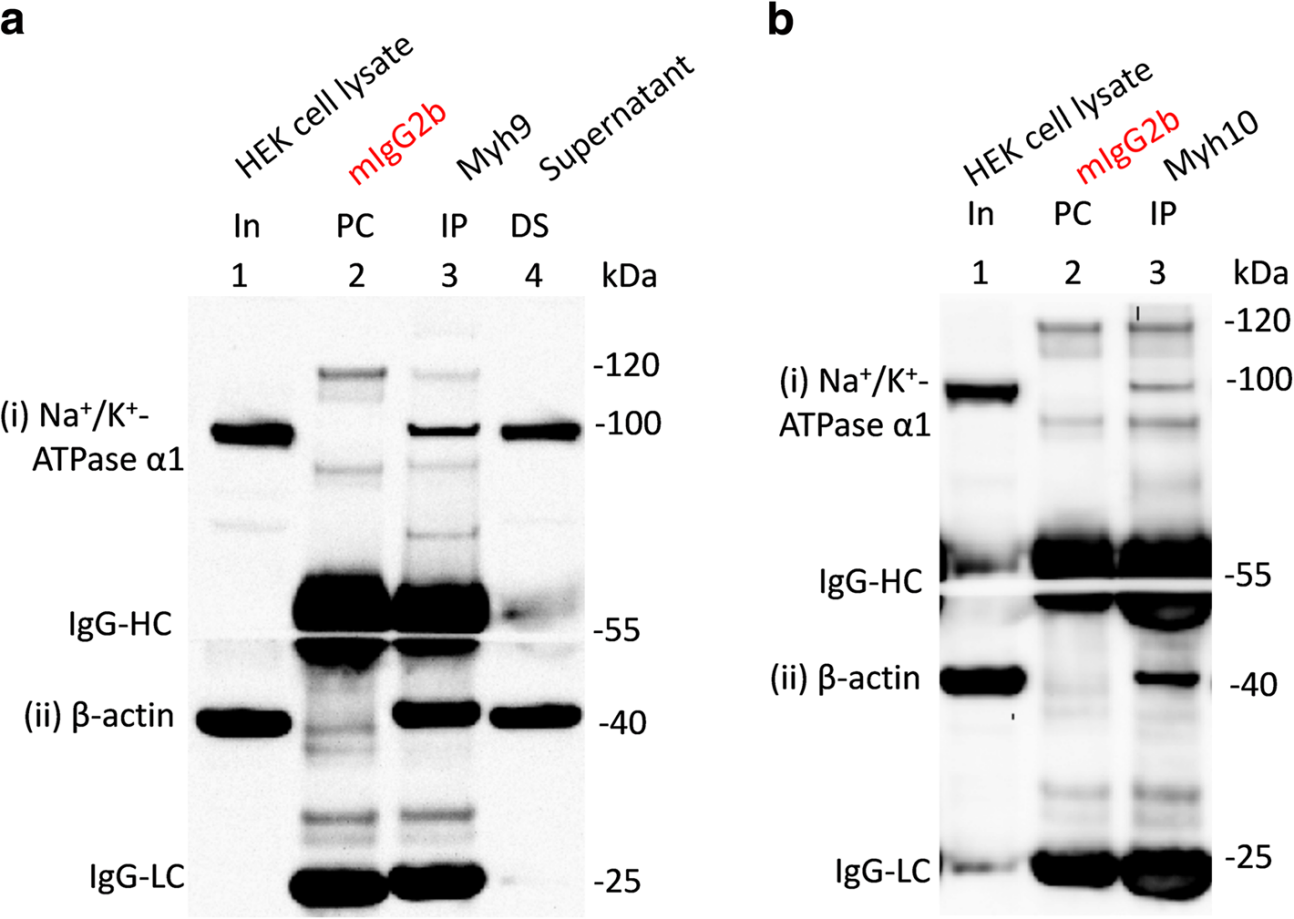
Interaction of non-muscle myosin heavy chains with Na^+^/K^+^-ATPase α1 subunits endogenously expressed in HEK293 cells. HEK293 cell lysates (In, lane 1 in **a** and **b**) were precleared (PC) with mouse IgG2b (PC, lane 2 in **a** and **b**) prior to immunoprecipitation (IP, lane 3) using antibodies for myh9 (**a**) and myh10 (**b**). Loading of PC complexes in the gel preceded those of the IP complexes. Immunoprecipitation of myh9 (**a**) and myh10 (**b**) led to the co-immunoprecipitation of Na^+^/K^+^ ATPase α1 subunits ((i) in **a** and **b**) and β-actin ((ii) in **a** and **b**) expressed in HEK293 cells. Na^+^/K^+^-ATPase α1 and β-actin immunoreactive signals in the depleted supernatant lane (DS, lane 4 in **a**) indicates that the ATPase survives the IP procedure. Denatured mouse IgG-HC and IgG-LC separated from their intact immunoglobulins (that is used for PC or IP) are seen in (**a** and **b**) as those blot sections were probed with mouse anti-β-actin antibodies

Antibodies already known to be suitable for immunoprecipitation (IP) were used. Antibodies, for which such information is not available, were considered suitable for IP assays when they would precipitate their cognate antigen and/or co-immunoprecipitate a known partner protein of their cognate antigens. Hence, for myosin antibodies we evaluated their ability to immunoprecipitate their respective cognate antigens and/or co-immunoprecipitate β-actin and/or MRLCs. This is because class II myosins (such as myh9, myh10, myh14; etc.) invariably interact with actins and MRLCs (Figs. 1, 2, 3 and Additional file 2: Figure S1).

**Fig. 4:**
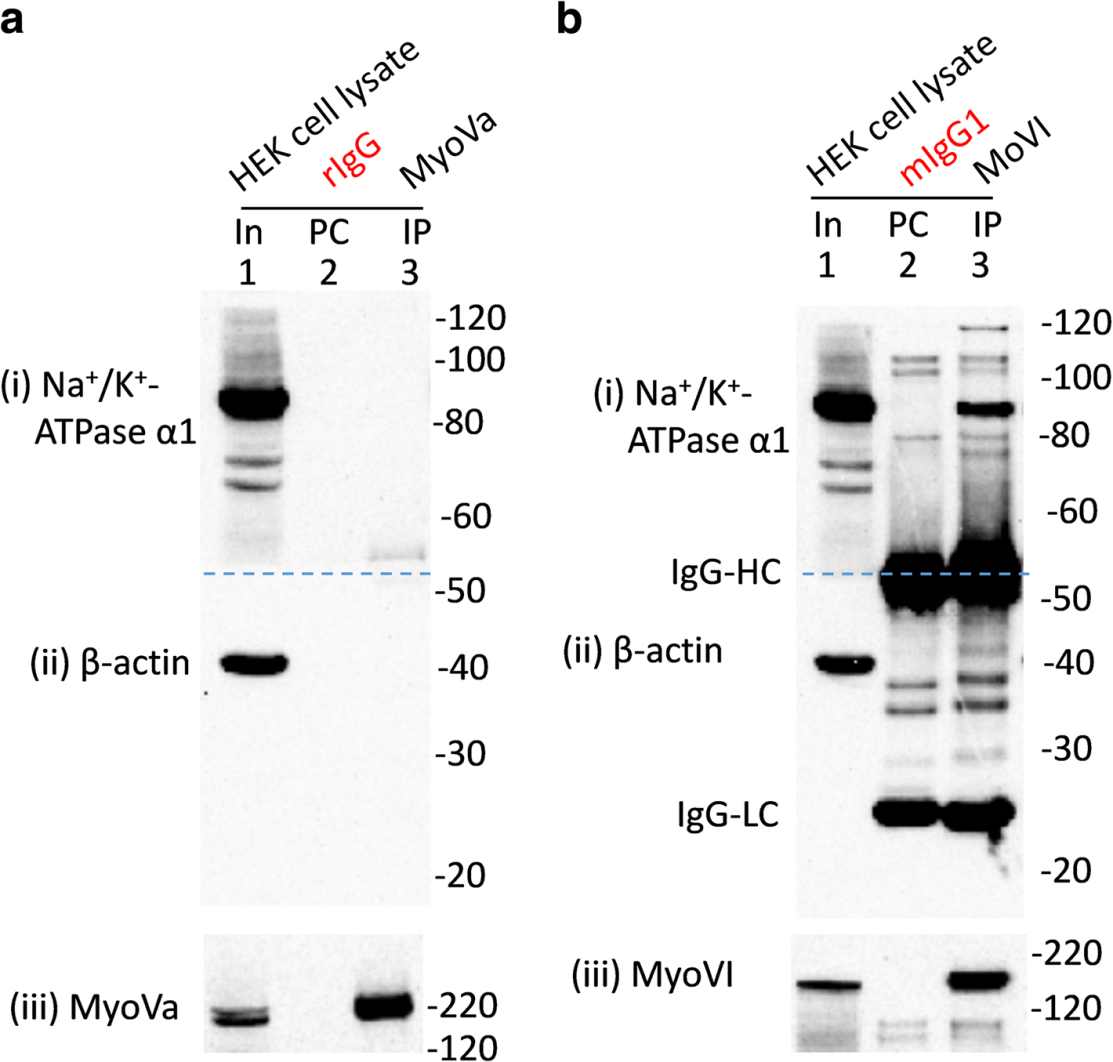
Interaction of myosin Va (myoVa) and myosin VI (myoVI) with Na^+^/K^+^-ATPase α1 subunits endogenously expressed in HEK293 cells. HEK293 cell lysates (In, lane 1 in **a** and **b**) were precleared (PC) with rabbit IgG (**a**) or mouse IgG1 (**b**) prior to immunoprecipitation (IP, lane 3) using antibodies for myoVa (**a**) or myoVI (**b**). Loading of PC complexes in the gel preceded those of the IP complexes. Immunoprecipitation of myoVI (**b** (iii)), but not those of myoVa (**a** (iii)), led to the co-immunoprecipitation of Na^+^/K^+^ ATPase α1 subunits ((i) in **a** and **b**) expressed in HEK293 cells. Neither myoVa nor myoVI co-immunoprecipitated β-actin ((ii) in **a** and **b**) expressed in HEK293 cells. Denatured mouse IgG-HC and IgG-LC separated from their intact immunoglobulins (that is used for PC or IP) are seen in B as the blot sections were probed with mouse anti- Na^+^/K^+^ ATPase α1 and anti-β-actin antibodies

**Fig. 5:**
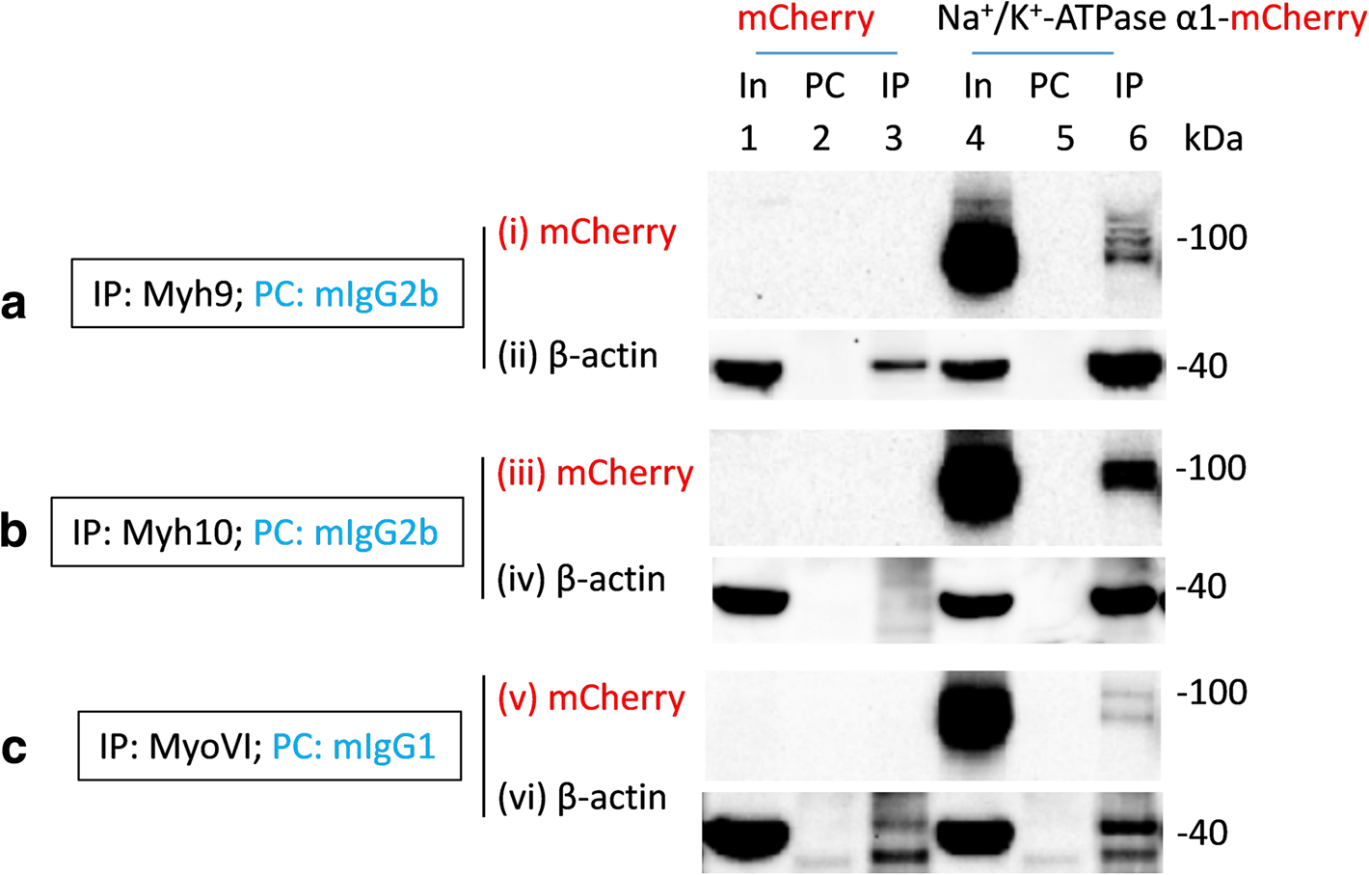
Multiple myosins co-immunoprecipitate recombinant Na^+^/K^+^-ATPase α1 subunits expressed in HEK293 cells. Lysates of HEK293 cells transiently transfected with mCherry (In, lane 1) or Na^+^/K^+^-ATPase α1 tagged with mCherry in the C-terminus (In, lane 4; Na^+^/K^+^ ATPase α1-mCherry) were precleared (PC) with mouse IgG2b antibodies (**a** and **b**; lanes 2 and 5) or mouse IgG1 antibodies (**c**; lanes 2 and 5) prior to immunoprecipitation (IP) using mouse anti-myh9 antibodies of the IgG2b isotypes (**a**; lanes 3 and 6), mouse anti-myh10 antibodies of the IgG2b isotypes (**b**; lanes 3 and 6) or mouse anti-myoVI antibodies of the IgG1 isotypes (**c**; lanes 3 and 6). Loading of PC complexes in the gel preceded those of the IP complexes. mCherry immunoreactive bands in lanes 4 and 6 but not in any other lanes indicated co-immunoprecipitation of recombinant Na^+^/K^+^-ATPase α1 subunits by myh9 (lane 6, panel (i)), myh10 (lane 6, panel (iii)) and myoVI (lane 6, panel (v)) from HEK293 cells transfected with Na^+^/K^+^-ATPase α1-mCherry plasmids but not from those transfected with mCherry. As expected β-actin (lanes 3 and 6 in panels (ii)) was co-immunoprecipitated with myh9. Myh10 and myoVI noticeably co-immunoprecipitated β-actin (lane 6 in (iv) and (vi)) from HEK293 cells overexpressing Na^+^/K^+^-ATPase α1 subunits but not from those overexpressing mCherry. Full length images of western blots are presented in Additional file 12: Figure S11, Additional file 13: Figure S12, Additional file 14: Figure S13)

First, we tested the ability of the myh9 (Abcam: ab55456) and myh10 (Abcam: ab684/3H2) antibodies to immunoprecipitate their cognate antigens and co-immunoprecipitate β-actin and/or MRLCs from HEK293 cells (Additional file 2: Figure S1). Both myh9 and myh10 antibodies immunoprecipitated their respective cognate antigens and co-immunoprecipitated β-actin and MRLCs from HEK293 cells. Myh9 appeared to co-immunoprecipitate β-actin better than myh10 from HEK293 cells which could be due to many factors [23] including the fact that myh10 antibodies are available as ascites and their use was determined empirically. However, both myh9 and myh10 antibodies pulled down β-actin from rat brain tissues very well (Fig. 1a ). We also assessed the ability of myh14 (a close homolog of myh9 and myh10 and the 3rd member of the class II NMHC), myoVa and myoVI to immunoprecipitate their respective cognate antigens and/or co-immunoprecipitate β-actin (Figs. 1b and 4). β-actin was co-immunoprecipitated with myh14, myoVa and myoVI to various degrees from rodent brain tissues (Fig. 1). We also observed that myoVa and myoVI antibodies would not or very poorly co-immunoprecipitate β-actin from HEK293 cells (Figs. 4 and 5). As expected the microtubule-based kinesin motor, KIF5B, did not co-immunoprecipitate β-actin from rat brain tissue lysates (Fig. 1a).

**Fig. 6:**
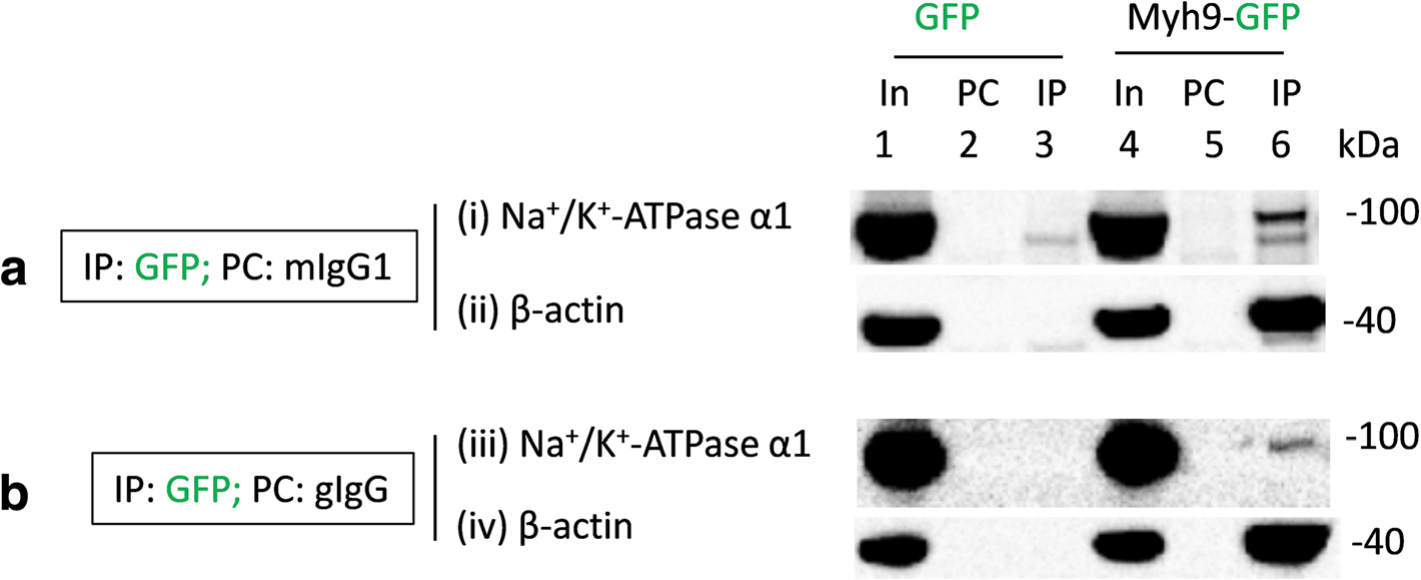
Recombinant myh9 co-immunoprecipitate Na^+^/K^+^-ATPase α1 subunits expressed in HEK293 cells. Lysates of HEK293 cells transiently transfected with GFP (In, lane 1) or myh9 tagged with GFP in the C-terminus (In, lane 4; myh9-GFP) were precleared (PC) with mouse IgG1 antibodies (**a**, lanes 2 and 5) or goat immunoglobulins (gIgG) (**b**, lanes 2 and 5) prior to immunoprecipitation (IP) using mouse anti-GFP antibodies of the IgG1 isotypes (**a**, lanes 3 and 6) or goat anti-GFP antibodies (**b**, lanes 3 and 6). Loading of PC complexes in the gel preceded those of the IP complexes. Na^+^/K^+^-ATPase α1 immunoreactive bands in lanes 1, 4 and 6 but not in any other lanes ((i) and (iii)) indicated co-immunoprecipitation of Na^+^/K^+^-ATPase α1 subunits from HEK293 cells transfected with myh9-GFP plasmids but not from those transfected with GFP plasmids. As expected immunoprecipitation using anti-GFP antibodies led to the co-immunoprecipitation of β-actin (lane 6 in (ii) and (iv)) from HEK293 cells transfected with myh9-GFP plasmids but not from those transfected with GFP plasmids. Immunoprecipitation using goat anti-GFP antibodies (**b**) led to a cleaner co-IP of Na^+^/K^+^-ATPase α1 subunits from HEK293 cells expressing myh9-GFP. Full length images of western blots are presented in Figure Additional file 4: Figure S3B (for part **a**) and Additional file 5: Figure S4B (for part **b**)

We also immunoprecipitated recombinant myh9 (Fig. 6, Additional file 3: Figure S2, Additional file 4: Figure S3B**,** Additional file 5: Figure S4 and Additional file 6: Figure S5), myh10 (Additional file 3: Figure S2, Additional file 4: Figure S3 and Additional file 6: Figure S5), myh14 (Additional file 4: Figure S3A, Additional file 5: Figure S4 and Additional file 7: Figure S6), myoVa (Additional file 8 Figure S7) and myoVI (Additional file 9: Figure S8) expressed in HEK293 cells to determine whether they would reflect the β-actin and/or MRLC binding abilities of myosins antibodies characterized earlier. Immunoprecipitation of recombinant myh9 (GFP tag either in the N- or C-terminus), myh10 (GFP tag either in the C-terminus) and myh14 (GFP tag in the C-terminus) using anti-GFP antibodies led to the co-immunoprecipitate β-actin and/or MRLCs from HEK293 cells. Neither recombinant myoVa nor myoVI (tagged with GFP in the C-terminus) would co-immunoprecipitate β-actin from HEK293 cells. These results recaptured the findings from myosin antibody-based assays that NMHC-IIs, but not myoVa nor myoVI, co-immunoprecipitate β-actin from HEK293 cells. These results also demonstrated that the GFP antibodies are suitable for use in IP assays.

The suitability of the mCherry antibodies (Abcam: ab183628) for use in IP assays was evaluated by immunoprecipitating mCherry tagged ankyrin-G (AnkG) (Additional file 10: Figure S9). Immunoprecipitation of AnkG-mCherry (i.e. AnkG tagged with mCherry in their C-terminus: AnkG270-mCherry = AnkG-mCherry) using mCherry antibodies led to the co-IP of Na^+^/K^+^-ATPase α1 subunits (a known partner of AnkG) from HEK293 cells. Also, AnkG270-mCherry, but not mCherry, IP led to the co-IP of Na_v_1.6 subunits (a known partner of AnkG) and Na^+^/K^+^-ATPase α1 subunits when HEK293 cells were co-expressing both AnkG270-mCherry and Na_v_1.6 subunits. These results also suggested that the mCherry antibodies are suitable for use in IP assays.

The suitability of anti-Na^+^/K^+^-ATPase α1 antibodies for use in IP assays was evaluated by determining their ability to immunoprecipitate their cognate antigens and/or co-immunoprecipitate β-actin (as Na^+^/K^+^-ATPase α1 subunits are known to interact with β-actin) (Figs. 2 and Additional file 11: Figure S10) [24]. As expected immunoprecipitation using Na^+^/K^+^-ATPase α1 antibodies (DSHB: a6F or Millipore: 05–369) led to the precipitation of its cognate antigens and co-IP of β-actin.

Thus, all the antibodies used in IP assays appeared to suitable for such purposes.

### Interaction of myosins with Na^+^/K^+^-ATPase α1 subunits expressed in the rodent brain or HEK293 cells

In this work we largely focus on the interaction of NMHC-IIs, myoVa and myoVI with Na^+^/K^+^-ATPase α1 subunits. It is because these myosins are well studied and heavily involved in the transport and trafficking of various cellular cargoes [11]. Non-muscle myosin II isoforms (i.e., myh9, myh10 and myh14), myoVa and myoVI were immunoprecipitated from adult rat brain tissue lysates to ascertain the potential in vivo interaction of these myosin motors with Na^+^/K^+^-ATPase α1 subunits (Fig. 1). Sodium/potassium ATPase α1 subunits were co-immunoprecipitated with myh9, myh10, myh14, myoVa and myoVI expressed in adult rat brain tissues (Fig. 1a and b). Kinesin-1 heavy chain (KIF5B) was immunoprecipitated to control for the co-IP Na^+^/K^+^-ATPase α1 subunits. Immunoprecipitation of KIF5B led to the co-immunoprecipitation of Na^+^/K^+^-ATPase α1 subunits expressed in rat brain tissues (Fig. 1a). This was not surprising given that kinesin light chain 2 (KLC2) is involved in the movement of Na^+^/K^+^-ATPase-containing vesicles [8].

We also investigated whether the cell background would have any effect on the interaction of NMHC-IIs, myoVa and myoVI with Na^+^/K^+^-ATPase α1 subunits. To this end we immunoprecipitated these myosins from HEK293 cells and probed for the co-immunoprecipitation Na^+^/K^+^-ATPase α1 subunits expressed there. Na^+^/K^+^-ATPase α1 subunits were co-immunoprecipitated with myh9, myh10, myh14 and myoVI expressed in HEK293 cells (Figs. 3 and 4). However, myoVa did not co-immunoprecipitate Na^+^/K^+^-ATPase α1 subunits expressed in HEK293 cells. These results are in agreement with the findings from the brain tissues except that myoVa could not co-immunoprecipitate Na^+^/K^+^-ATPase α1 subunits expressed in HEK293 cells.

Reciprocally, we also wanted to determine whether the immunoprecipitation of Na^+^/K^+^-ATPase α1 subunits from adult rat brain tissues would lead to the co-immunoprecipitation of any of the myosins targeted in this study (Fig. 1b). Results indicated that Na^+^/K^+^-ATPase α1 subunits expressed in the rat brain could co-immunoprecipitate myh9, myh10 and myoVa but not myoVI (Fig. 2). We also observed that IP of Na^+^/K^+^-ATPase α1 subunits led to the co-IP of MRLCs expressed in rat brain tissues (Fig. 2; panel (iv)). MRLCs are associated with NMHC-IIs and other myosins (smooth muscle myosin/Myh11, myosin 15/Myo15, myosin 18A/Myo18A, myosin 19/Myo19) expressed in the nervous tissues [25–27]. It is possible that co-IP of MRLCs with Na^+^/K^+^-ATPase α1 subunits is as a result of Na^+^/K^+^-ATPase α1 subunits pulling down class NMHC-IIs and other myosins expressed in rat brain tissues. Alternatively, it is plausible that Na^+^/K^+^-ATPase α1 subunits might be interacting with MRLCs directly or indirectly via other proteins.

Similarly, we wanted to know whether the immunoprecipitation of Na^+^/K^+^-ATPase α1 subunits from HEK293 cells would lead to the co-immunoprecipitation of any of the myosins targeted in this study (Additional file 11: Figure S10). To our surprise, Na^+^/K^+^-ATPase α1 subunits could not co-immunoprecipitate myh9, myh10, myoVa or myoVI expressed in HEK293 cells though anti-Na^+^/K^+^-ATPase α1 antibodies immunoprecipitated Na^+^/K^+^-ATPase α1 subunits and co-immunoprecipitated β-actin from HEK293 cells (not all data are shown; Additional file 11: Figure S10).

### Interaction of myosins with recombinant Na^+^/K^+^-ATPase α1 subunits

Based on our earlier findings we investigated whether myh9, myh10 or myoVI would co-immunoprecipitate recombinant Na^+^/K^+^-ATPase α1 subunits expressed in HEK293 cells. To this end, we overexpressed mCherry (as a control) and Na^+^/K^+^-ATPase α1 subunits tagged with mCherry in their C-terminus (i.e., Na^+^/K^+^-ATPase α1-mCherry) in HEK293 cells (Fig. 5, Additional file 12: Figure S11, Additional file 13: Figure S12 and Additional file 14: Figure S13). Both myh9 and myh10 co-immunoprecipitated recombinant Na^+^/K^+^-ATPase α1 subunits (i.e., Na^+^/K^+^-ATPase α1-mCherry), but not mCherry, expressed in HEK293 cells. Similarly, myoVI also co-immunoprecipitated Na^+^/K^+^-ATPase α1-mCherry, but not mCherry, expressed in HEK293 cells (Fig. 5c ). The co-immunoprecipitation signals for Na^+^/K^+^-ATPase α1-mCherry subunits by myh9, myh10 or myoVI revealed multiple mCherry immunoreactive bands which could be, among others, as a result of overexpression, degradation, propeptide cleavage and post-translational modifications of recombinant Na^+^/K^+^-ATPase α1 subunits expressed in HEK293 cells [28]. We also observed significant co-immunoprecipitation of β-actin by myh10 and myoVI (lane 6, panel (vi), Fig. 5c) only in the presence of heterologously expressed Na^+^/K^+^-ATPase α1 subunits in HEK293 cells. This was surprising as myh10 and myoVI antibodies poorly co-immunoprecipitate β-actin from HEK293 cells. Hence, the β-actin co-immunoprecipitation seen in lane 6 of panels (iv) and (vi) (Fig. 5c) appears to be as a result of overexpression of Na^+^/K^+^-ATPase α1 subunits.

### Interaction of recombinant myosins with Na^+^/K^+^-ATPase α1 subunits

We also determined whether recombinant myosins would co-immunoprecipitate Na^+^/K^+^-ATPase α1 subunits expressed in HEK293 cells. To this end, we overexpressed GFP (as a control), GFP-myh9 and GFP-myh10 (GFP tag in their N-terminus) in HEK293 cells (Additional file 3: Figure. S2). Results indicated a lack of or very poor co-immunoprecipitation of endogenous Na^+^/K^+^-ATPase α1 subunits as a result of immunoprecipitation of GFP-myh9 or GFP-myh10 heterologously expressed in HEK293 cells (Additional file 3: Figure S2). As expected GFP-myh9, not GFP, co-immunoprecipitated β-actin expressed in HEK293 cells. However, immunoprecipitation of GFP-myh10 using mouse anti-GFP antibodies did not lead to co-immunoprecipitation of β-actin expressed in HEK293 cells. Use of a pan-Na^+^/K^+^-ATPase α subunit antibody (Santa Cruz Biotechnology: sc-58,628/clone M7-PB-E9) for immunoblotting did not alter the outcome seen with the use of anti-Na^+^/K^+^-ATPase α1 subunit antibodies (DSHB) (Additional file 3: Figure S2B). We reasoned the fusion of the GFP tag in the N-terminus of myh9 and myh10 could be partly responsible for this outcome.

**Fig. 7:**
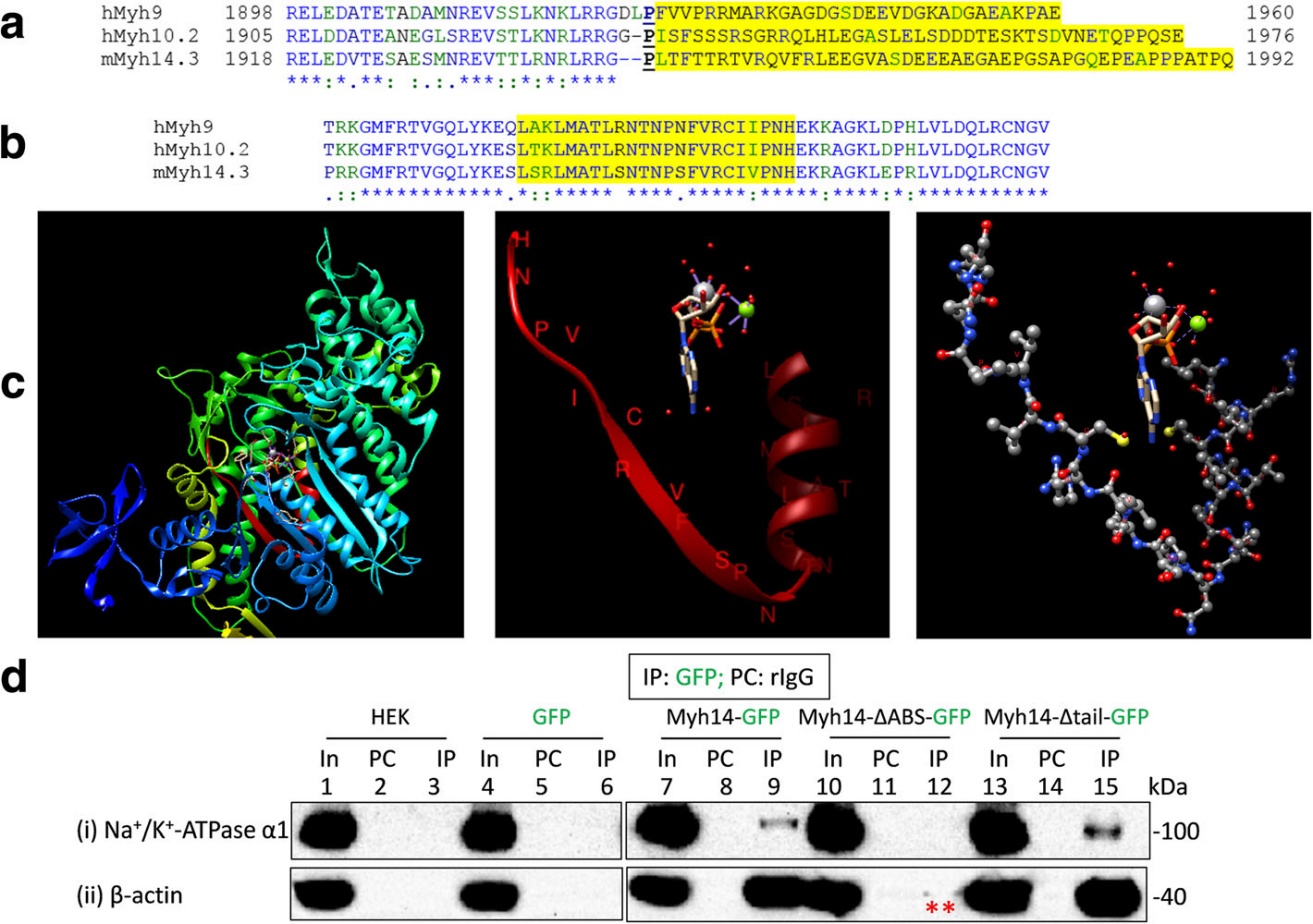
Interaction of actin-binding-site-less (ΔABS) and tail-less (Δtail) NMHC-IIs with Na^+^/K^+^-ATPase α1 subunits expressed in HEK293 cells. **a** Tail-less NMHC-IIs were made by deleting the tail regions, yellow shaded, of human (h) myh9 (i.e., hMyh9 AAs: 1928–1960), human (h) myh10 isoform 2 (i.e., hMyh10.2 AAs: 1934–1976) and mouse (m) myh14 isoform 3 (i.e., mMyh14.3 AAs:1946–1992) following a conserved proline residue (bold and underlined) where the numbers indicate the position of the amino acids in the WT constructs. **b** Actin-binding-site-less (ΔABS) NMHC-IIs were made by deleting a 23 amino acids (AAs) segment, yellow shaded, of hMyh9 (i.e., AAs: 654–676), hMyh10.2 (i.e., AAs: 661–683) and mMyh14.3 (i.e., AAs: 674–696). For both (**a**) and (**b**) symbols below sequences indicate fully (*), strongly (:) or weakly (.) conserved residues, and the lack of a symbol indicates amino acid divergence. **c** The actin binding site (ABS) of human myh14. The 3-D coordinates of human myh14 was retrieved from protein data bank (PDB: 5I4E). Structural features of myh14 was visualized and analyzed using UCSF Chimera (http://www.cgl.ucsf.edu/chimera/). Left panel shows the relative position of the ABS (red ribbon), nucleotide analog (adenosine diphosphate vanadate (ADP.VO4): ball and stick model) binding site and N-terminal SH3 domain (deep blue) in the motor domain of human myh14 (5I4E.pdb). The center and right panel show the relative position of the ABS (red ribbon; and ball and stick model) with respect to the position of the adenosine diphosphate vanadate (ADP.VO4) and magnesium co-factor (green ball) in the motor domain of myh14. **d** Tail-less (Δtail) but not actin-binding-site-less (ΔABS) myh14 co-immunoprecipitate Na^+^/K^+^-ATPase α1 subunits expressed in HEK293 cells. Lysates of non-transfected HEK293 cells (In; 1) or HEK293 cells transiently transfected with GFP (In; 4), myh14-GFP (In; 7), myh14-ΔABS-GFP (In; 10), or myh14-Δtail-GFP (In; 13) plasmids (where the GFP tag is in their C-terminus) were precleared with rabbit IgG (PC; lanes 2, 5, 8, 11 and 14) prior to immunoprecipitation using rabbit anti-GFP antibodies (IP; lanes 3, 6, 9, 12 and 15). Loading of PC complexes in the gel preceded those of the IP complexes. Presence of Na^+^/K^+^-ATPase α1 immunoreactive bands in lanes 1, 4, 7, 9, 10, 13 and 15 and absence of any Na^+^/K^+^-ATPase α1 immunoreactive bands in lanes 2, 3, 5, 6, 8, 11, 12 and 14 (in (i)) indicated co-immunoprecipitation of Na^+^/K^+^-ATPase α1 subunits from HEK293 cells transfected with myh14-GFP or myh14-Δtail-GFP plasmids but not from non-transfected HEK293 cells or those transfected with GFP or myh14-ΔABS-GFP plasmids. While myh14-ΔABS-GFP showed almost complete loss of actin binding (indicated with double asterisk ‘*’; lane 12, panel (ii)) both myh14-GFP and myh14-Δtail-GFP co-immunoprecipitated β-actin (lane 9 and lane 15 in panel (ii) respectively). Full length images of western blots are presented in Additional file 7: Figure S6 B

Next, we immunoprecipitated myh9, myh10 or myh14 tagged with GFP in the C-terminus (i.e., myh9-GFP, myh10-GFP or myh14-GFP respectively) using anti-GFP antibodies of mouse, rabbit and/or goat origin to investigate whether these recombinant myosins, not GFP, upon heterologous expression would co-immunoprecipitate Na^+^/K^+^-ATPase α1 subunits expressed in HEK293 cells (Figs. 6, 7 and Additional file 4: Figure S3, Additional file 5: Figure S4, Additional file 6: Figure S5 and Additional file 7: Figure S6). Immunoprecipitation of myh9-GFP or myh14-GFP using the same mouse anti-GFP antibodies led to the co-immunoprecipitation of Na^+^/K^+^-ATPase α1 subunits and β-actin (Additional file 4: Figure S3). However, immunoprecipitation of myh9-GFP (Fig. 6b and Additional file 5: Figure S4), myh10-GFP (Additional file 6: Figure S5) and myh14-GFP (Fig. 7 and Additional file 7: Figure S6) using goat anti-GFP antibodies or rabbit anti-GFP antibodies led to a much cleaner co-immunoprecipitation of Na^+^/K^+^-ATPase α1 subunits expressed in HEK293 cells.

**Fig. 8:**
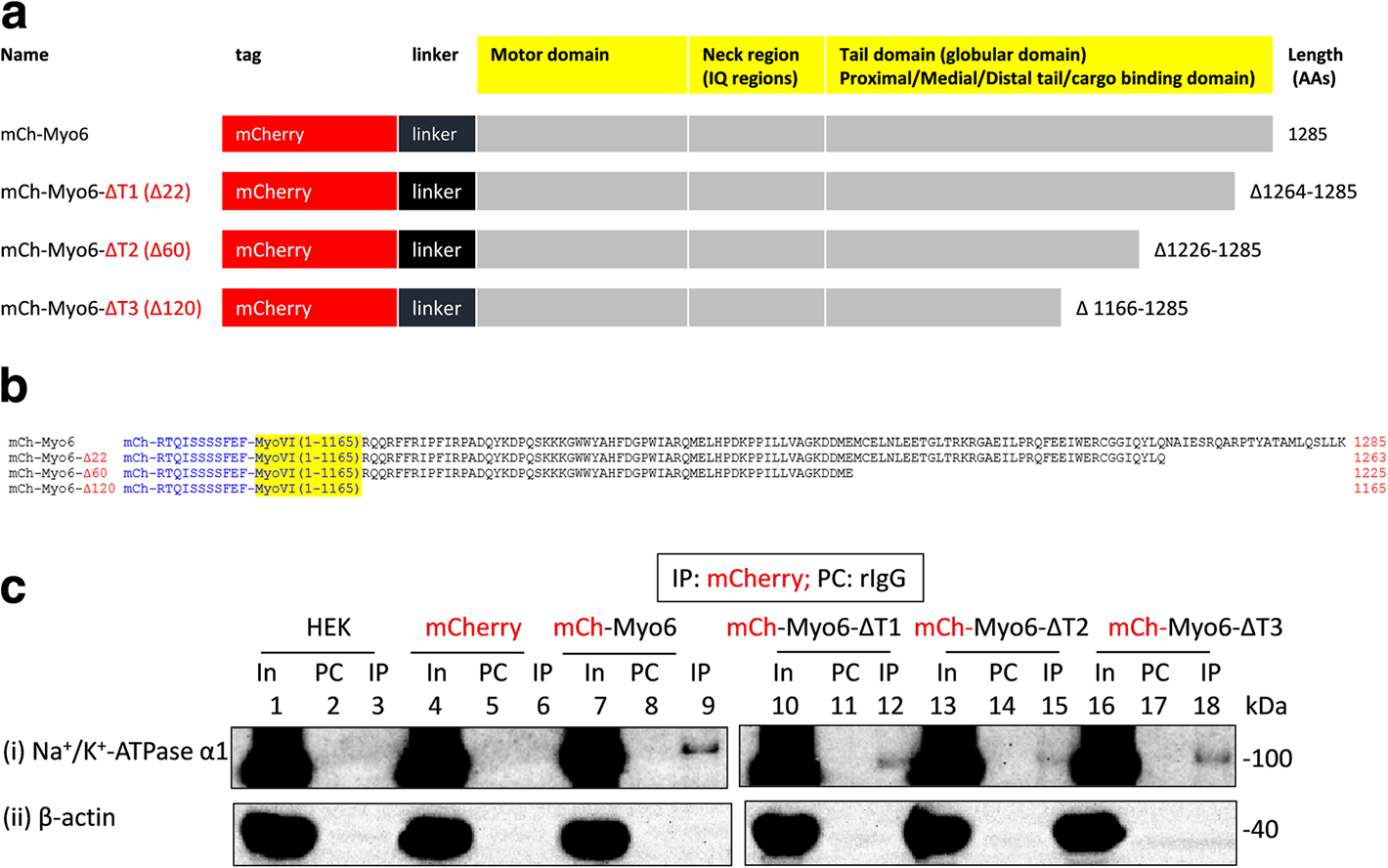
Interaction of full length or tail-less (ΔT) recombinant myo6 with Na^+^/K^+^-ATPase α1 subunits expressed in HEK293 cells. **a** and **b** Schematic representation of myo6 constructs. All the myo6 constructs possessed a short amino acid linker of 11 AAs (shown in blue colored letters in **b**) between the mCherry tag (N-terminal) and coding sequences for myo6. mCherry tagged full length myo6 constructs (i.e., mCherry-Myo6) were truncated by 22 AAs (i.e., Δ1264–1285 = mCherry-myoVI-ΔT1), 60 AAs (i.e., Δ1226–1285 = mCherry-myoVI-ΔT2) and 120 AAs (i.e., Δ1166–1285 = mCherry-myoVI-ΔT3) from the C-terminal end where the numbers indicate the amino acid positions in the WT myo6. **c** Lysates of non-transfected HEK293 cells (In; 1) or HEK293 cells transiently transfected with mCherry (In; 4), mCherry-myo6 (In; 7), mCherry-myo6-ΔT1 (In; 10), mCherry-myo6-ΔT2 (In; 13) or mCherry-myo6-ΔT3 (In; 16) plasmids (where mCherry tag is in the N-terminus) were precleared with rabbit IgG (PC; lanes 2, 5, 8, 11, 14 and 17) prior to immunoprecipitation using rabbit anti-mCherry antibodies (IP; lanes 3, 6, 9, 12, 15 and 18). Loading of PC complexes in the gel preceded those of the IP complexes. Presence of Na^+^/K^+^-ATPase α1 immunoreactive bands in lanes 1, 4, 7, 9, 10, 12, 13, 15, 16 and 18 in (i) and absence of any Na^+^/K^+^-ATPase α1 immunoreactive bands in lanes 2, 3, 5, 6, 8, 11, 14 and 17 in (i) indicated co-immunoprecipitation of Na^+^/K^+^-ATPase α1 subunits from the IP of recombinant mCherry-myo6, mCherry-myo6-ΔT1, mCherry-myo6-ΔT2 or mCherry-myo6-ΔT3 but not from those of mCherry thus confirming interaction between myo6 and Na^+^/K^+^-ATPase α1 subunits which is not perturbed due to loss of tail regions in myo6. Recombinant mCherry-Myo6, mCherry-myo6-ΔT1, mCherry-myo6-ΔT2 or mCherry-myo6-ΔT3 could not co-immunoprecipitate β-actin (i.e., lanes 9, 12, 15 and 18 in (ii)) from HEK293 cells. Full length images of western blots are presented in Additional file 15: Figure S14

We employed an approach similar to the one described above to study the interaction of recombinant myoVI with Na^+^/K^+^-ATPase α1 subunits expressed in HEK293 cells. Hence, we tagged myoVI with GFP in the C-terminus (i.e., myoVI-GFP) and expressed them in HEK293 cells (Additional file 9: Figure S8). Surprisingly, immunoprecipitation of recombinant myoVI using rabbit or goat anti-GFP antibodies did not lead to the co-immunoprecipitation of Na^+^/K^+^-ATPase α1 subunits expressed in HEK293 cells (Additional file 9: Figure S8). As a recourse, we tagged myoVI with mCherry in the N-terminus (i.e., mCherry-myoVI) and expressed them in HEK293 cells. mCherry-myoVI, but not mCherry, immunoprecipitation led to the co-immunoprecipitation of Na^+^/K^+^-ATPase α1 subunits expressed in HEK293 cells (Fig. 8).

### Tail-less myosins interact with Na^+^/K^+^-ATPase α1 subunits

Myosins employ various adaptor proteins to transport their cargoes (i.e., myosin-adaptor-cargo) [29, 30]. The adaptor proteins typically interact with the tail regions of various myosins. Hence myosins possess cargo binding domains (CBD) in their tail regions. Therefore, we wanted to know whether the tail regions of NMHC-IIs (i.e., myh9, myh10, myh14) and myoVI are involved in their interaction with Na^+^/K^+^-ATPase α1 subunits.

We deleted the tail regions of NMHC-IIs (i.e., 33, 43 and 47 AAs from their C-terminal ends in myh9, myh10 and myh14 respectively) to make myh9-Δtail-GFP, myh10-Δtail-GFP or myh14-Δtail-GFP (Fig. 7a). Then we overexpressed and immunoprecipitated tail-less (Δtail) recombinant NMHC-IIs (i.e., myh9-Δtail-GFP, myh10-Δtail-GFP or myh14-Δtail-GFP) and probed for their ability to interact with Na^+^/K^+^-ATPase α1 subunits expressed in HEK293 cells. Tail-less myh9 (i.e., myh9-Δtail-GFP), myh10 (i.e., myh10-Δtail-GFP) or myh14 (i.e., myh14-Δtail-GFP) were able to co-immunoprecipitate Na^+^/K^+^-ATPase α1 subunits expressed in HEK293 cells (Fig. 7d and Additional file 5: Figure S4, Additional file 6: Figure S5 and Additional file 7: Figure S6). These results indicated that regions other than the tail ends are probably involved in the interaction of NMHC-IIs with Na^+^/K^+^-ATPase α1 subunits expressed in HEK293 cells.

Next, we wanted to know whether tail-less (ΔT) myoVI would interact with Na^+^/K^+^-ATPase α1 subunits expressed in HEK293 cells (Fig. 8). The C-terminal end (or tail region) of myoVI possesses many sites for interaction with various adaptor proteins and/or cargoes [29, 30]. Hence, first we made and expressed a recombinant myoVI that lacked 22 AAs (i.e., i.e., mCherry-Myo6-ΔT1) from its C-terminal end and examined its ability to interact with Na^+^/K^+^-ATPase α1 subunits expressed in HEK293 cells (Fig. 8). Immunoprecipitation of mCherry-myoVI-ΔT1, but not mCherry, led to the co-immunoprecipitation of Na^+^/K^+^-ATPase α1 subunits expressed in HEK293 cells (Fig. 8c). Then we made two additional myoVI deletion constructs which lacked either 60 AAs (i.e., mCherry-Myo6-ΔT2) or 120 AAs (i.e., mCherry-Myo6-ΔT3) from their C-terminal ends. Like mCherry-Myo6-ΔT1, both mCherry-Myo6-ΔT2 and mCherry-Myo6-ΔT3 co-immunoprecipitated Na^+^/K^+^-ATPase α1 subunits expressed in HEK293 cells (Fig. 8c). Hence, tail-less myo6, like those of tail-less NMHC-IIs, interact with Na^+^/K^+^-ATPase α1 subunits and regions aside their tail regions appear to be involved in the interaction of myoVI with Na^+^/K^+^-ATPase α1 subunits.

### The actin binding sites (ABS) of NMHC-IIs influence their interaction with Na^+^/K^+^-ATPase α1 subunits

Previous results indicated that tail-less NMHC-IIs interact with Na^+^/K^+^-ATPase α1 subunits. These results indicated that region(s) other than the C-terminal tail ends in NMHC-IIs is/are important for their interaction with Na^+^/K^+^-ATPase α1 subunits. Hence a non-tail region such as motor domain (site for ATP and actin binding), neck region (the site for light chain binding) or a conserved AA motif in these domains could be involved in the interaction of NMHC-IIs with Na^+^/K^+^-ATPase α1 subunits. This is plausible as the tails are divergent and a common or conserved mechanism appear to be involved in the interaction of NMHC-IIs with Na^+^/K^+^-ATPase α1 subunits. Additionally, we knew (and observed) that Na^+^/K^+^-ATPase α1 subunits co-immunoprecipitate β-actin [24] and NMHC-IIs (i.e., myh9, myh10 or myh14), native or recombinant, also co-immunoprecipitate β-actin. Hence, we wanted to determine whether the conserved actin binding site (ABS) of NMHC-IIs (Fig. 7b) are important in their interaction with Na^+^/K^+^-ATPase α1 subunits.

To this end, we made and expressed recombinant myh9, myh10 or myh14 lacking 22 AAs (involved in actin binding) from their motor head regions (i.e., myh9-ΔABS-GFP, myh10-ΔABS-GFP and myh14-ΔABS-GFP) in HEK293 cells (Fig. 7b and Additional file 5: Figure S4**,** Additional file 6: Figure S5 and Additional file 7: Figure S6) [18, 19]. Immunoprecipitation of myh9-ΔABS-GFP (Additional file 5: Figure S4), myh10-ΔABS-GFP (Additional file 6: Figure S5) or myh14-ΔABS-GFP (Fig. 7d and Additional file 7: Figure S6) led to reduced or complete lack of co-IP of Na^+^/K^+^-ATPase α1 subunits from HEK293 cells. As expected, β-actin co-IP was drastically reduced or eliminated from these ABS-less recombinant NMHC-IIs. Hence it appears that the ABS in NMHC-IIs is involved in the interaction of NMHC-IIs with Na^+^/K^+^-ATPase α1 subunits.

## Discussion

Class II myosins, traditionally known as conventional myosins, are expressed in striated muscles (i.e., myh1, myh2, myh3, myh4; etc.), cardiac muscles (i.e., myh6, myh7, and myh7B), smooth muscles (myh11) and non-muscles (i.e., myh9, myh10, and myh14) [31]. The non-muscle myosin II heavy chains (NMHC-IIs) (i.e., three of them: NMHC-IIA/myh9, NMHC-IIB/myh10, and NMHC-IIC/myh14) are widely expressed, as their name suggests, in non-muscle cells including neurons, microglia, podocytes; etc. [32–35]. They are also expressed in cardiomyocytes and at low levels in muscle cells [32, 34]. In this work we show that all the three NMHC-IIs (i.e., myh9, myh10 and myh14) interact with Na^+^/K^+^-ATPase α1 subunits expressed in the rodent brain and HEK293 cells (Table 1). These results may not be surprising as NMHC-IIs are homologous protein molecules possibly possessing similar structural and functional features for their interaction with Na^+^/K^+^-ATPase α1 subunits although they are engaged in unique biological functions at distinct cellular locations [36–40]. The interaction of NMHC-IIs with Na^+^/K^+^-ATPase α1 subunits are consistent with previous findings that NMHC-IIs interact with various proteins such as C-X-C chemokine receptor type 4 (CXCR4) [41], collagen receptor DDR1 (discoidin domain receptor 1) [42], Ins (1,4,5)P_3_ receptor [43], epidermal growth factor receptor (EGFR) [44], N-Methyl-d-aspartate (NMDA) receptors [45], α-amino-3-hydroxy-5-methyl-4-isoxazolepropionic acid (AMPA) receptor [46], the pore-forming subunit of P/Q-type calcium channels (Ca_v_2.1) [47], battenin/juvenile Batten disease protein (Cln3: a lysosomal membrane protein) [48]; etc. A recent report implicates non-muscle myosin II (NM-II) in the sorting and post-Golgi dendritic trafficking of Kv2.1 channels [49]. Hence our results possibly indicate that NMHC-IIs could be involved in the trafficking of Na^+^/K^+^-ATPase α1 subunits. These results are also suggesting that other class II myosins (such as myh1, myh3, myh6, myh7, myh14; etc.), which are similar to NMHC-IIs in their structural configurations, could be involved in the transport and/or trafficking of Na^+^/K^+^-ATPase α1 subunits in cells (such as cardiomyocytes, skeletal muscles, smooth muscles; etc.) where both molecules are expressed.

**Table 1:** Summary of interaction of various myosins with Na^+^/K^+^-ATPase α1 subunits obtained via immunoprecipitation (IP) and co-IP assay

	Immunoprecipitation (IP) of native myosins		IP of recombinant myosins	IP of native Na^+^/K^+^ -ATPase α1	
Interaction/Tissue or cell types	Brain tissues	HEK293 cells	HEK293 cells	Brain tissues	HEK293 cells
Myh9:: Na^+^/K^+^-ATPase α1	Yes	Yes	Yes	Yes	No
Myh10::Na^+^/K^+^-ATPase α1	Yes	Yes	Yes	Yes	No
Myh14::Na^+^/K^+^-ATPase α1	Yes	–	Yes	–	No
MyoVa::Na^+^/K^+^-ATPase α1	Yes	No	No	Yes	No
MyoVI::Na^+^/K^+^-ATPase α1	Yes	Yes	Yes	No	No

‘-‘indicates unavailability of data

Positive interaction of NMHC-IIs with Na^+^/K^+^-ATPase α1 subunits prompted us to investigate whether non-class II myosins (i.e., unconventional myosins) would interact with Na^+^/K^+^-ATPase α1 subunits. Myosin Va (myoVa) immediately caught our attention. In alveolar epithelial cells it is involved in restraining Na^+^/K^+^-ATPase-containing vesicles within intracellular pools and overexpression of dominant-negative myoVa or knockdown with specific shRNA increased the average speed and distance traveled by the Na^+^/K^+^-ATPase-containing vesicles [9]. These results suggested that myoVa might be interacting with Na^+^/K^+^-ATPase α1 subunits expressed in alveolar epithelial cells. Our data indicate that myoVa interact with Na^+^/K^+^-ATPase α1 subunits expressed in the brain tissues (Figs. 1 and 2, Table 1) but not in HEK293 cells. This is possibly an indication that myoVa interact with Na^+^/K^+^-ATPase α1 subunits in a tissue specific manner and one or more molecular components required for their interaction in native tissues might not be present in HEK293 cells. Nonetheless our results are consistent with earlier observation that myoVa is involved in the trafficking of membrane proteins such as AMPA receptors; glucose transporter type 4 (GLUT-4); etc. [9, 50, 51]. These results also suggest that myoVa might be involved in the anterograde trafficking of Na^+^/K^+^-ATPase α1 subunits because of its known role in such processes [9, 51–56].

Our results also indicated that myoVI, another unconventional myosin, interact with Na^+^/K^+^-ATPase α1 subunits (Table 1). It is consistent with previous reports that myoVI interact or associate with various proteins and is involved in their transport and trafficking [9, 20, 57–60]. For example, myoVI is implicated in the trafficking and sorting of a transmembrane receptor (PlexinD1), transporter (GLUT1), cotransporters (NaPi2a and NaPi2c), transmembrane conductance regulator (CFTR); etc. [58]. It is possible that myoVI might be involved in the retrograde trafficking of Na^+^/K^+^-ATPase α1 subunits because of its known role in endocytosis, autophagy and trafficking of ubiquitinated cargoes [57, 58].

We observed that Na^+^/K^+^-ATPase α1 subunits interact with the myosin regulatory light chains (MRLCs; also known as regulatory light chain 20 (RLC20)). This is consistent with previous observations that myosin light chains are important for interaction of NMHC-IIs with their partner proteins. Both the heavy and light chains of NMHC-IIB interact with the cytoplasmic C-terminal region of the Ca(v)2.1 subunit of P/Q-type calcium channels [47]. Similarly, the interaction of NMHC-IIs and EGFR requires the regulatory light chain 20 (RLC20) of NMHC-IIs [44]. These results indicate that the neck region, the site for MRC or RLC20 binding, of NMHC-IIs could be important for their interaction with partner proteins including Na^+^/K^+^-ATPase α1 subunits. As a whole the observations that Na^+^/K^+^-ATPase α1 subunits interact with multiple myosins and MRLCs are consistent with earlier observations that they interact and/or co-localize with various proteins including atypical sodium channel (Na_x_: SCN7A, SCN6A), water channel aquaporin 4 (AQP4), ionotropic glutamatergic AMPA receptors (AMPARs), glutamate transporter (GLAST and GLT-1), glycine transporter (GlyT2), STIM1(stromal interaction molecule 1)-POST (partner of STIM1) complex, follistatin-like 1 (FSTL1), Polycystin-1 (PC-1); etc. [61–69].

We observed that tail-less NMHC-IIs and myoVI interact with Na^+^/K^+^-ATPase α1 subunits. These results indicated that region(s) other than the C-terminal tail ends in NMHC-IIs is/are important for their interaction with Na^+^/K^+^-ATPase α1 subunits. Hence a non-tail region such as motor domain, neck region or a conserved AA motif in these domains could be involved in the interaction of NMHC-IIs with Na^+^/K^+^-ATPase α1 subunits. Consistent with these ideas we show that NMHC-IIs lacking their actin binding sites, which are conserved in myosins, show reduced or null interaction with Na^+^/K^+^-ATPase α1 subunits. Hence, actin binding sites of NMHC-IIs are possibly involved in their interaction with Na^+^/K^+^-ATPase α1 subunits. These results also indicate that actin might be involved in the interaction of NMHC-IIs with Na^+^/K^+^-ATPase α1 subunits. Moreover, both NMHC-IIs and Na^+^/K^+^-ATPase α1 subunits directly interact with actin. Therefore, it is plausible that a tripartite interaction of Na^+^/K^+^-ATPase, β-actin and myosin could be occurring where (monomeric or polymeric) actin might serve as a link between Na^+^/K^+^-ATPase α1 subunits and myosin (i.e., Na^+^/K^+^-ATPase::actin:myosin complex).

Alternatively, it is possible that myosins (such as myh9, myh10, myh14, myoVa and myoVI) are interacting with Na^+^/K^+^-ATPase α1 subunits using adaptor or partner proteins. This idea of a myosin-adaptor- Na^+^/K^+^-ATPase complex may not be farfetched as Na^+^/K^+^-ATPase α1 subunits contain multiple structural motifs that enable them to interact with various soluble, membrane and structural proteins such as ankyrins, BiP, calnexin, cofilin, adducin, actin; etc. [2, 3, 70–73]. It appears that NMHC-IIs could be using a common set of adaptors for their interaction with Na^+^/K^+^-ATPase α1 subunits though alternative use of unique adaptors can’t be discounted.

In this work we provide evidence that actins and myosins interact with Na^+^/K^+^-ATPase α1 subunits. The actin-myosin network is involved in short range trafficking whereas the microtubule-kinesin network is involved in long range cellular trafficking [52]. Moreover, in neurons microtubules extend along the full length of the axon and transect actins both in the soma and axon. Hence it appears that kinesins could play a role in concert with myosins to localize Na^+^/K^+^-ATPase α1 subunits (sodium pump) to far off places from the cell body. Overall, our data support a model in which the actin-myosin network is involved in the trafficking of sodium pumps in neuronal and non-neuronal tissues.

## Additional files

Additional file 1: **Table S1.** Antibodies used for immunoprecipitation (IP) assays. **Table S2.** Antibodies used for immunoblotting. **Table S3.** Various features of the cDNA constructs used in the study. (DOCX 21 kb)

Additional file 2: **Figure S1.** Anti-myh9 and anti-myh10 antibodies immunoprecipitate their cognate antigens and/or co-immunoprecipitate partner proteins (such as β-actin and/or MRLCs) of their cognate antigens from HEK293 cells. Lysates of non-transfected HEK293 cells (In, lane 1 in A and B) were precleared (PC) with mouse IgG2b isotypes (mIgG2b; lane 2 in A and B) prior to immunoprecipitation (IP) using antibodies for myh9 (lane 3 in A) and myh10 (lane 3 B) of mIgG2b isotypes. IP complexes in the gel were loaded following the loading of their respective PC complexes. Myh9 immunoreactive bands in lane 3 of panel (i) in A and myh10 immunoreactive bands in lane 3 of panel (i) in B indicated immunoprecipitation of myh9 and myh10 by their respective antibodies. Presence of β-actin immunoreactive bands in the IP lanes of A (ii) and B (ii) indicated co-immunoprecipitation of it by myh9 and myh10 from non-transfected HEK293 cells. Both myh9 and myh10 also co-immunoprecipitated MRCLs (panel (iii) of A and B) from HEK293 cells. An asterisk (‘*’) in A and B indicates lack of detection of MRLCs in the input samples. Myh9 or myh10 immunoreactive bands in the depleted supernatant lanes (DS, lane 4 in panel (i) in A and B) indicate that both Mg^2+^-ATPases survive the IP procedure. Mouse IgG-HC and IgG-LC (panel (ii) in A and B) separated from their intact immunoglobulins (that is used for PC or IP) upon denaturation could be seen as this section of the blot is probed with mouse anti-β-actin antibodies. (TIF 1319 kb)

Additional file 3: **Figure S2.** Lack of co-immunoprecipitation of Na^+^/K^+^-ATPase α1 subunits by recombinant myh9 or myh10 tagged with GFP-in their N-termini. Lysates of non-transfected HEK293 cells (In; lane 1 in B) or HEK293 cells transiently transfected with GFP (In; lane 4 in A and B), GFP-myh9 (In; lane 7 in A and B) or GFP-myh10 (In; lane 10 in A and B) plasmids were precleared with mouse IgG1 isotypes (PC; lanes 2, 5, 8 and 11 in A or B) prior to immunoprecipitation using mouse anti-GFP antibodies (IP; lanes 3, 6, 9 and 12; Abcam: ab1218) of the IgG1 isotypes. Loading of PC complexes in the gel preceded those of the IP complexes. Na^+^/K^+^-ATPase α1 (Abcam: ab7671) immunoreactive bands in the input lanes 4, 7 and 10 but not in the PC or IP lanes 5, 6, 8, 9, 11 and 12 (A (i)) or Na^+^/K^+^-ATPase α (pan- Na^+^/K^+^-ATPase α) immunoreactive bands (Santa Cruz Biotechnology: sc-58,628) in the input lanes 1, 4, 7 and 10 but not in the PC or IP lanes 2, 3, 5, 6, 8, 9, 11 and 12 (B (ii)) indicated lack of co-immunoprecipitation of Na^+^/K^+^-ATPase α (or α1) subunits by N-terminally GFP tagged myh9 or myh10 expressed in HEK293 cells. GFP-myh9 (but not GFP-myh10) co-immunoprecipitated β-actin (lanes 9 vs. 12 in panel (ii) of A and B). Stripping and staining the uppermost section of the blot with rabbit anti-GFP antibodies indicated successful immunoprecipitation of GFP-myh9 (lane 9 in (iii) in A) and GFP-myh10 (lane 12 in (iii) in A) from HEK293 cell lysates. Denatured mouse IgG-HC and/or IgG-LC (iii) separated from their intact immunoglobulins (used in PC or IP reactions) are seen as the blot section is probed with mouse anti-β-actin antibodies. (TIF 2367 kb)

Additional file 4: **Figure S3.** Co-immunoprecipitation of Na^+^/K^+^-ATPase α1 subunits by C-terminally GFP tagged myh14 or myh9. Lysates of non-transfected HEK293 cells (In; lane 1 in A) or HEK293 cells transiently transfected with GFP (In; lane 4 in A and B), myh14-GFP (In; lane 7 in A) or myh9-GFP (In; lane 7 in B) plasmids were precleared with mouse IgG1 isotypes (PC; lanes 2, 5 and 8 in A and B) prior to immunoprecipitation using mouse anti-GFP antibodies (IP; lanes 3, 6 and 9 in A and B; Abcam: ab1218) of the IgG1 isotypes. Loading of PC complexes in the gel preceded those of the IP complexes. Na^+^/K^+^-ATPase α1 (Abcam: ab7671) immunoreactive bands in IP lane 9 (denoted by asterisk “*” in (i) in A and B) but not in any other IP or PC lanes indicated co-immunoprecipitation of Na^+^/K^+^-ATPase α1 subunits by C-terminally GFP tagged myh14 or myh9 expressed in HEK293 cells. Both myh14-GFP and myh9-GFP (but not GFP) co-immunoprecipitated β-actin (lane 9 in (ii) in A and B). Myh9-GFP (but not GFP) also co-immunoprecipitated MRLC (lane 9 in (iii) in B). Denatured mouse IgG-HC and/or IgG-LC separated from their intact immunoglobulins (used in PC or IP reactions) are observed as those blot sections are probed with mouse antibodies (for Na^+^/K^+^-ATPase α1, β-actin and/or MRLCs). Part of S3B is presented in Fig. 6a. (TIF 2846 kb)

Additional file 5: **Figure S4.** Interaction of full length, actin binding site less (ΔABS) or tail-less (Δtail) recombinant myh9 with Na^+^/K^+^-ATPase α1 subunits and β-actin expressed in HEK293 cells*.* Lysates of non-transfected HEK293 cells (In; lane 1 in A, B and C) or HEK293 cells transiently transfected with GFP (In; lane 4 in A, B and C), myh9-GFP (In; lane 7 in A, B and C), myh9-ΔABS-GFP (In; lane 10 in C) or myh9-Δtail-GFP (In; lane 13 in C) plasmids (where the GFP tag is in their C-terminus) were precleared with rabbit IgG (PC; lanes 2, 5 and 8 in A) or goat IgG (PC; lanes 2, 5, 8, 11 and 14 in B or C) prior to immunoprecipitation using rabbit anti-GFP antibodies (IP; lanes 3, 6 and 9 in A) or goat anti-GFP antibodies (IP; lanes 3, 6, 9, 12 and 15 in B or C). Presence of obvious Na^+^/K^+^-ATPase α1 immunoreactive bands in lanes 1, 4, 7, 9, 10, 13 and 15 in A, B or C; greatly reduced Na^+^/K^+^-ATPase α1 immunoreactive bands in lane 12 in C; and absence of any Na^+^/K^+^-ATPase α1 immunoreactive bands in lanes 2, 3, 5, 6, 8, 11 and 14 in A, B or C indicated co-immunoprecipitation of Na^+^/K^+^-ATPase α1 subunits (panel (i)) from HEK293 cells transfected with myh9-GFP, myh9-ΔABS-GFP or myh9-Δtail-GFP plasmids thus confirming interaction between myh9 and Na^+^/K^+^-ATPase α1 subunits which is almost abrogated due to loss of actin binding site but not the tail regions in myh9. Myh9-GFP and myh9-Δtail-GFP, but not GFP, co-immunoprecipitated β-actin (lanes 9 and 15 respectively in panel (ii)). There was almost total loss of actin binding upon deletion of the actin binding site in myh9 (panel (ii), lane 12). Part of S4B is presented in Fig. 6b. (TIF 4636 kb)

Additional file 6: **Figure S5.** Interaction of full length, actin-binding-site-less (ΔABS) or tail-less (Δtail) recombinant myh10 with Na^+^/K^+^-ATPase α1 subunits and β-actin expressed in HEK293 cells*.* Lysates of HEK293 cells transiently transfected with myh10-GFP (In; lane 1 in A, B and C), myh10-ΔABS-GFP (In; lane 4 in A, B and C) or myh10-Δtail-GFP (In; lane 7 in A, B and C) plasmids (where the GFP tag is in their C-terminus) were precleared with rabbit IgG (PC; lanes 2, 5 and 8 in A) or goat IgG (PC; lanes 2, 5 and 8 in B and C) prior to immunoprecipitation using rabbit anti-GFP antibodies (IP; lanes 3, 6 and 9 in A) or goat anti-GFP antibodies (IP; lanes 3, 6 and 9 in B and C). Loading of PC complexes in the gel preceded those of the IP complexes. Presence of Na^+^/K^+^-ATPase α1 immunoreactive bands in lanes 1, 3, 4, 7 and 9 (panel (i) in A, B and C) and greatly reduced or lack of presence of Na^+^/K^+^-ATPase α1 immunoreactive bands in lane 5 (panel (i) in A, B and C) indicated co-immunoprecipitation of Na^+^/K^+^-ATPase α1 subunits from HEK293 cells transfected with myh10-GFP or myh10-Δtail-GFP plasmids but not from those transfected with myh10-ΔABS-GFP plasmids thus confirming interaction between myh10 and Na^+^/K^+^-ATPase α1 subunits which is eliminated due to loss of actin binding site but not the tail regions in myh10. Myh10-GFP co-immunoprecipitated β-actin (lane 3 in (ii) in A, B and C) and there was complete loss of actin binding upon deletion of its actin binding site (lane 6 in (ii) in A, B and C). Tail-less myh10 also co-immunoprecipitated β-actin (lane 9 in (ii) in A, B and C). Control experiments for non-transfected HEK293 cells or HEK293 cells transiently transfected with GFP are done previously. (TIF 3566 kb)

Additional file 7: **Figure S6.** Interaction of full length, actin binding site less (ΔABS) or tail-less (Δtail) recombinant myh14 with Na^+^/K^+^-ATPase α1 subunits and β-actin expressed in HEK293 cells*.* Lysates of non-transfected HEK293 cells (In; lane 1 in A and B) or HEK293 cells transiently transfected with GFP (In; lane 4 in A and B), myh14-GFP (In; lane 7 in A and B), myh14-ΔABS-GFP (In; lane 10 in A and B) or myh14-Δtail-GFP (In; lane 13 in A and B) plasmids (where the GFP tag is in their C-terminus) were precleared with rabbit IgG (PC; lanes 2, 5, 8 11 and 14 in A) or goat IgG (PC; lanes 2, 5, 8, 11 and 14 in B) prior to immunoprecipitation using rabbit anti-GFP antibodies (IP; lanes 3, 6, 9, 12 and 15 in A) or goat anti-GFP antibodies (IP; lanes 3, 6, 9, 12 and 15 in B). Presence of obvious Na^+^/K^+^-ATPase α1 immunoreactive bands in lanes 1, 4, 7, 9, 10, 13 and 15 in A or B; absence of Na^+^/K^+^-ATPase α1 immunoreactive bands in lane 12 in A or B; and absence of any Na^+^/K^+^-ATPase α1 immunoreactive bands in lanes 2, 3, 5, 6, 8, 11 and 14 in A or B indicated co-immunoprecipitation of Na^+^/K^+^-ATPase α1 subunits (panel (i)) from HEK293 cells transfected with myh14-GFP, myh14-ΔABS-GFP or myh14-Δtail-GFP plasmids thus confirming interaction between myh14 and Na^+^/K^+^-ATPase α1 subunits which is abrogated due to loss of actin binding site but not the tail regions in myh14. Myh14-GFP and myh14-Δtail-GFP co-immunoprecipitated β-actin (lanes 9 and 15 respectively in panel (ii) in A and B). There was almost total loss of actin binding upon deletion of the actin binding site in myh14 (panel (ii), lane 12 in A and B). Part of S6B is presented in Fig. 7d. (TIF 4560 kb)

Additional file 8: **Figure S7.** Lack of co-immunoprecipitation of Na^+^/K^+^-ATPase α1 subunits and β-actin by recombinant myoVa. Lysates of HEK293 cells transiently transfected with GFP or myoVa-GFP plasmids were precleared with rabbit IgG (PC; lanes 2 and 5 in A) or goat IgG (PC; lanes 2 and 5 in B) prior to immunoprecipitation using rabbit anti-GFP antibodies (IP; lanes 3 and 6 in A) or goat rabbit anti-GFP antibodies (IP; lanes 3 and 6 in B). Loading of PC complexes in the gel preceded those of the IP complexes. Presence of Na^+^/K^+^-ATPase α1 immunoreactive bands in lanes 1 and 4, and absence of any Na^+^/K^+^-ATPase α1 immunoreactive bands in lanes 2, 3, 5 and 6 (panel (i) in A and B) indicated lack of co-immunoprecipitation of Na^+^/K^+^-ATPase α1 subunits from HEK293 cells transfected with myoVa-GFP. MyoVa-GFP did not co-immunoprecipitate β-actin (lane 6 in (ii) in A and B) from HEK293 cells. Staining the blots with mouse anti-GFP antibodies (NeuroMab: 75–131) indicated successful immunoprecipitation of GFP (lane 3 in (iii) in A and B) and myoVa-GFP (lane 6 in (iv) in B) from HEK293 cell lysates. (TIF 1906 kb)

Additional file 9: **Figure S8.** Lack of co-immunoprecipitation of Na^+^/K^+^-ATPase α1 subunits and β-actin by recombinant myoVI. Lysates of HEK293 cells transiently transfected with GFP or myoVI-GFP plasmids were precleared with rabbit IgG (PC; lanes 2 and 5 in A) or goat IgG (PC; lanes 2 and 5 in B) prior to immunoprecipitation using rabbit anti-GFP antibodies (IP; lanes 3 and 6 in A) or goat anti-GFP antibodies (IP; lanes 3 and 6 in B). Loading of PC complexes in the gel preceded those of the IP complexes. Presence of Na^+^/K^+^-ATPase α1 immunoreactive bands in lanes 1 and 4, and absence of any Na^+^/K^+^-ATPase α1 immunoreactive bands in lanes 2, 3, 5 and 6 (panel (i) in A and B) indicated lack of co-immunoprecipitation of Na^+^/K^+^-ATPase α1 subunits from HEK293 cells transfected with myoVI-GFP. MyoVI-GFP did not co-immunoprecipitate β-actin (lane 6 in (ii) in A and B) from HEK293 cells. Staining the blots with mouse anti-GFP antibodies (NeuroMab: 75–131) indicated successful immunoprecipitation of GFP (lane 3 in (iii) in A and B) and myoVI-GFP (lane 6 in (iv) in A and B) from HEK293 cell lysates. The input signal for myoVI-GFP (indicated with an asterisk ‘*’) appears to be lost during stripping and/or staining with anti-GFP antibodies (lane 4 in (iv)). (TIF 2117 kb)

Additional file 10: **Figure S9.** mCherry antibodies are suitable for use in IP assay. Lysates of HEK293 cells transiently transfected with mCherry plasmids (In, lane 1) or ankyrin-G-mCherry plasmids (mCherry fused to the C-terminus: AnkG-mCh) (In, lane 4) or co-transfected with both AnkG-mCherry and Na_v_1.6 plasmids (In, lane 7) were precleared (PC) with rabbit IgG (PC; lanes 2, 5 and 8) prior to immunoprecipitation using rabbit anti-mCherry antibodies (IP; lanes 3, 6 and 9). Loading of PC complexes in the gel preceded those of the IP complexes. Pan-Na_v_α immunoreactive bands (panel (i)) in IP lane 9 but not in other IP (i.e., 3 and 6) or PC lanes (2 and 5) indicated co-IP of Na_v_1.6 subunits (IP, lane 9) by recombinant ankyrin-G from HEK293 cells co-transfected with both ankyrin-G and Na_v_1.6 subunits but not from HEK293 cells transfected with mCherry or AnkG-mCherry. Similarly, Na^+^/K^+^-ATPase α1 immunoreactive bands (panel (ii)) in lanes 1, 4, 6, 7 and 9 (but not in lanes 2, 3, 5 and 8) indicated co-IP of Na^+^/K^+^-ATPase α1 subunits (i.e., lanes 6 and 9) by recombinant ankyrin-G from HEK293 cells transfected with recombinant AnkG alone or along with Na_v_1.6 subunits but not from HEK293 cells transfected with mCherry thus confirming interaction between AnkG and Na^+^/K^+^-ATPase α1 subunits. Also, mCherry immunoreactive bands in lanes 4, 6, 7 and 9 (panel (iii)) indicated IP of AnkG-mCherry by mCherry antibodies. (TIF 1055 kb)

Additional file 11: **Figure S10.** Na^+^/K^+^-ATPase α1 subunits could not co-immunoprecipitate myh9 expressed in HEK293 cells. (A) Immunoprecipitation of Na^+^/K^+^-ATPase α1 subunits expressed in HEK293 cells. HEK293 cell lysates (In, lane 1) were precleared (PC, lane 2) with mouse IgG2a isotypes prior to immunoprecipitation (IP, lane 3) using mouse anti-Na^+^/K^+^-ATPase α1 antibodies (DSHB: a6F) of the IgG2a isotypes. Na^+^/K^+^ ATPase α1 immunoreactive bands in lanes 1, 3 and 5 but not in lane 2 (i) indicated immunoprecipitation of Na^+^/K^+^-ATPase α1 subunits expressed in HEK293 cells by the antibody in use. Na^+^/K^+^-ATPase α1 immunoreactive band was also observed in the depleted supernatant lane (DS, lane 4). As expected Na^+^/K^+^-ATPase α1 subunits also co-immunoprecipitated β-actin from HEK293 cells (iii). Denatured mouse IgG-HC (ii) and IgG-LC (iv) separated from their intact immunoglobulins (that is used for PC or IP) are seen as the blot was probed with mouse antibodies (for Na^+^/K^+^-ATPase α1 or β-actin). (B) and (C). Lack of co-immunoprecipitation of myh9 by Na^+^/K^+^-ATPase α1 subunits. HEK293 cell lysates (In, lane 1) were precleared (PC, lane 2) with indicated immunoglobulin isotypes (PC; mIgG2a in B and mIgG1 in C) prior to immunoprecipitation (IP, lane 3) using mouse anti-Na^+^/K^+^-ATPase α1 antibodies (DSHB: a6F in B and EMD Millipore; clone C464.6 in C). Antibodies for Na^+^/K^+^-ATPase α1 subunits could not co-immunoprecipitate myh9 (lane 3, panel (i) in B or C) though they could co-immunoprecipitate β-actin (lane 3, panel (iii) in B or C) and immunoprecipitate their cognate antigens (lane 3, panel (ii) in B or C) from HEK293 cells. (TIF 2570 kb)

Additional file 12: **Figure S11.** Myh9 co-immunoprecipitate recombinant Na^+^/K^+^-ATPase α1 subunits expressed in HEK293 cells. Part of S11 is presented in Fig. 5a. (TIF 2388 kb)

Additional file 13: **Figure S12.** Myh10 co-immunoprecipitate recombinant Na^+^/K^+^-ATPase α1 subunits expressed in HEK293 cells. Part of S12 is presented in Fig. 5b. (TIF 2619 kb)

Additional file 14: **Figure S13.** MyoVI co-immunoprecipitate recombinant Na^+^/K^+^-ATPase α1 subunits expressed in HEK293 cells. Part of S13 is presented in Fig. 5c. (TIF 2302 kb)

Additional file 15: **Figure S14.** Interaction of full length or tail-less (ΔT) recombinant myo6 with Na^+^/K^+^-ATPase α1 subunits expressed in HEK293 cells. Part of S14 is presented in Fig. 8c. (TIF 6409 kb)

## Abbreviations

AA: Amino acid
DS: Depleted supernatant
ELC: Essential light chain
GFP: Green fluorescent protein
gIgG: Goat immunoglobulins
HEK293 cells: Human embryonic 293 cells
IB: Immunoblot
IgG: Immunoglobulin
IgG-HC: Immunoglobulin heavy chain
IgG-LC: Immunoglobulin light chain
In: Lysate input
IP: Immunoprecipitation
KIF5B: kinesin family member 5B = kinesin-1 heavy chain
mCh: mCherry
mIgG1: Mouse immunoglobulin isotype 1
mIgG2a: Mouse immunoglobulin isotype 2a
mIgG2b: Mouse immunoglobulin isotype 2b
MM: Protein molecular weight marker
Myh: Myosin heavy chain
Myh10: Myosin heavy chain 10
Myh14: Myosin heavy chain 14
Myh9: Myosin heavy chain 9
MyoVa: Myo5a = Myosin Va
MyoVI: Myo6 = Myosin VI
Na^+^/K^+^-ATPase alpha 1: Sodium/potassium ATPase α1
NMHC: Non-muscle myosin heavy chain
PC: Pre-clear/immunoprecipitation using an isotype antibody
rIgG: Rabbit immunoglobulins
RLC: Regulatory light chain
WT: Wild type

## References

[1] Clausen MV, Hilbers F, Poulsen H (2017). The structure and function of the Na,K-ATPase Isoforms in Health and Disease. Front Physiol.

[2] Xie Z, Cai T (2003). Na+-K+−-ATPase-mediated signal transduction: from protein interaction to cellular function. Mol Interv.

[3] Tian J, Cai T, Yuan Z, Wang H, Liu L, Haas M, Maksimova E, Huang XY, Xie ZJ (2006). Binding of Src to Na+/K+-ATPase forms a functional signaling complex. Mol Biol Cell.

[4] Reinhard L, Tidow H, Clausen MJ, Nissen P (2013). Na(+),K (+)-ATPase as a docking station: protein-protein complexes of the Na(+),K (+)-ATPase. Cell Mol Life Sci.

[5] Blanco G, Mercer RW (1998). Isozymes of the Na-K-ATPase: heterogeneity in structure, diversity in function. Am J Phys.

[6] Forrest MD (2014). The sodium-potassium pump is an information processing element in brain computation. Front Physiol.

[7] Ikeda K, Onimaru H, Kawakami K (1666). Knockout of sodium pump alpha3 subunit gene (Atp1a3(−/−)) results in perinatal seizure and defective respiratory rhythm generation. Brain Res.

[8] Trejo HE, Lecuona E, Grillo D, Szleifer I, Nekrasova OE, Gelfand VI, Sznajder JI (2010). Role of kinesin light chain-2 of kinesin-1 in the traffic of Na,K-ATPase-containing vesicles in alveolar epithelial cells. FASEB J.

[9] Lecuona E, Minin A, Trejo HE, Chen J, Comellas AP, Sun H, Grillo D, Nekrasova OE, Welch LC, Szleifer I (2009). Myosin-Va restrains the trafficking of Na+/K+-ATPase-containing vesicles in alveolar epithelial cells. J Cell Sci.

[10] Hartman MA, Spudich JA (2012). The myosin superfamily at a glance. J Cell Sci.

[11] Kneussel M, Wagner W (2013). Myosin motors at neuronal synapses: drivers of membrane transport and actin dynamics. Nat Rev Neurosci.

[12] Chinthalapudi K, Heissler SM, Preller M, Sellers JR, Manstein DJ. Mechanistic insights into the active site and allosteric communication pathways in human nonmuscle myosin-2C. Elife. 2017;6 10.7554/eLife.32742 PMC5749951 29256864

[13] Kondo T, Okada M, Kunihiro K, Takahashi M, Yaoita Y, Hosoya H, Hamao K (2015). Characterization of myosin II regulatory light chain isoforms in HeLa cells. Cytoskeleton (Hoboken).

[14] Li C, Wen A, Shen B, Lu J, Huang Y, Chang Y (2011). FastCloning: a highly simplified, purification-free, sequence- and ligation-independent PCR cloning method. BMC Biotechnol.

[15] Wei Q, Adelstein RS (2000). Conditional expression of a truncated fragment of nonmuscle myosin II-A alters cell shape but not cytokinesis in HeLa cells. Mol Biol Cell.

[16] Golomb E, Ma X, Jana SS, Preston YA, Kawamoto S, Shoham NG, Goldin E, Conti MA, Sellers JR, Adelstein RS (2004). Identification and characterization of nonmuscle myosin II-C, a new member of the myosin II family. J Biol Chem.

[17] Breckenridge MT, Dulyaninova NG, Egelhoff TT (2009). Multiple regulatory steps control mammalian nonmuscle myosin II assembly in live cells. Mol Biol Cell.

[18] Maruta S, Homma K (1998). A unique loop contributing to the structure of the ATP-binding cleft of skeletal muscle myosin communicates with the actin-binding site. J Biochem.

[19] Suzuki R, Nishi N, Tokura S, Morita F (1987). F-actin-binding synthetic heptapeptide having the amino acid sequence around the SH1 cysteinyl residue of myosin. J Biol Chem.

[20] Arden SD, Tumbarello DA, Butt T, Kendrick-Jones J, Buss F (2016). Loss of cargo binding in the human myosin VI deafness mutant (R1166X) leads to increased actin filament binding. Biochem J.

[21] Leterrier C, Vacher H, Fache MP, d'Ortoli SA, Castets F, Autillo-Touati A, Dargent B (2011). End-binding proteins EB3 and EB1 link microtubules to ankyrin G in the axon initial segment. Proc Natl Acad Sci U S A.

[22] Barry J, Gu Y, Jukkola P, O'Neill B, Gu H, Mohler PJ, Rajamani KT, Gu C (2014). Ankyrin-G directly binds to kinesin-1 to transport voltage-gated Na+ channels into axons. Dev Cell.

[23] Kelley CA, Sellers JR, Gard DL, Bui D, Adelstein RS, Baines IC (1996). Xenopus nonmuscle myosin heavy chain isoforms have different subcellular localizations and enzymatic activities. J Cell Biol.

[24] Cantiello HF (1995). Actin filaments stimulate the Na(+)-K(+)-ATPase. Am J Phys.

[25] Bird JE, Takagi Y, Billington N, Strub MP, Sellers JR, Friedman TB (2014). Chaperone-enhanced purification of unconventional myosin 15, a molecular motor specialized for stereocilia protein trafficking. Proc Natl Acad Sci U S A.

[26] Guzik-Lendrum S, Heissler SM, Billington N, Takagi Y, Yang Y, Knight PJ, Homsher E, Sellers JR (2013). Mammalian myosin-18A, a highly divergent myosin. J Biol Chem.

[27] Lu Z, Ma XN, Zhang HM, Ji HH, Ding H, Zhang J, Luo D, Sun Y, Li XD (2014). Mouse myosin-19 is a plus-end-directed, high-duty ratio molecular motor. J Biol Chem.

[28] Fuller W, Tulloch LB, Shattock MJ, Calaghan SC, Howie J, Wypijewski KJ (2013). Regulation of the cardiac sodium pump. Cell Mol Life Sci.

[29] Li J, Lu Q, Zhang M (2016). Structural basis of cargo recognition by unconventional Myosins in cellular trafficking. Traffic.

[30] Buss F, Kendrick-Jones J (2011). Multifunctional myosin VI has a multitude of cargoes. Proc Natl Acad Sci U S A.

[31] Sellers JR (2000). Myosins: a diverse superfamily. Biochim Biophys Acta.

[32] Redowicz MJ (2007). Unconventional myosins in muscle. Eur J Cell Biol.

[33] Lofgren M, Ekblad E, Morano I, Arner A (2003). Nonmuscle myosin motor of smooth muscle. J Gen Physiol.

[34] Tullio AN, Accili D, Ferrans VJ, Yu ZX, Takeda K, Grinberg A, Westphal H, Preston YA, Adelstein RS (1997). Nonmuscle myosin II-B is required for normal development of the mouse heart. Proc Natl Acad Sci U S A.

[35] Janssen S, Gudi V, Prajeeth CK, Singh V, Stahl K, Heckers S, Skripuletz T, Pul R, Trebst C, Tsiavaliaris G, Stangel M (2014). A pivotal role of nonmuscle myosin II during microglial activation. Exp Neurol.

[36] Ma X, Kawamoto S, Hara Y, Adelstein RS (2004). A point mutation in the motor domain of nonmuscle myosin II-B impairs migration of distinct groups of neurons. Mol Biol Cell.

[37] Rubio MD, Johnson R, Miller CA, Huganir RL, Rumbaugh G (2011). Regulation of synapse structure and function by distinct myosin II motors. J Neurosci.

[38] Rex CS, Gavin CF, Rubio MD, Kramar EA, Chen LY, Jia Y, Huganir RL, Muzyczka N, Gall CM, Miller CA (2010). Myosin IIb regulates actin dynamics during synaptic plasticity and memory formation. Neuron.

[39] Ma X, Jana SS, Conti MA, Kawamoto S, Claycomb WC, Adelstein RS (2010). Ablation of nonmuscle myosin II-B and II-C reveals a role for nonmuscle myosin II in cardiac myocyte karyokinesis. Mol Biol Cell.

[40] Vicente-Manzanares M, Ma X, Adelstein RS, Horwitz AR (2009). Non-muscle myosin II takes Centre stage in cell adhesion and migration. Nat Rev Mol Cell Biol.

[41] Rey M, Vicente-Manzanares M, Viedma F, Yanez-Mo M, Urzainqui A, Barreiro O, Vazquez J, Sanchez-Madrid F (2002). Cutting edge: association of the motor protein nonmuscle myosin heavy chain-IIA with the C terminus of the chemokine receptor CXCR4 in T lymphocytes. J Immunol.

[42] Huang Y, Arora P, McCulloch CA, Vogel WF (2009). The collagen receptor DDR1 regulates cell spreading and motility by associating with myosin IIA. J Cell Sci.

[43] Hours MC, Mery L (2010). The N-terminal domain of the type 1 ins(1,4,5)P3 receptor stably expressed in MDCK cells interacts with myosin IIA and alters epithelial cell morphology. J Cell Sci.

[44] Kim JH, Wang A, Conti MA, Adelstein RS (2012). Nonmuscle myosin II is required for internalization of the epidermal growth factor receptor and modulation of downstream signaling. J Biol Chem.

[45] Bu Y, Wang N, Wang S, Sheng T, Tian T, Chen L, Pan W, Zhu M, Luo J, Lu W (2015). Myosin IIb-dependent regulation of actin dynamics is required for N-methyl-D-aspartate receptor trafficking during synaptic plasticity. J Biol Chem.

[46] Ryu J, Liu L, Wong TP, Wu DC, Burette A, Weinberg R, Wang YT, Sheng M (2006). A critical role for myosin IIb in dendritic spine morphology and synaptic function. Neuron.

[47] Marqueze-Pouey B, Martin-Moutot N, Sakkou-Norton M, Leveque C, Ji Y, Cornet V, Hsiao WL, Seagar M (2008). Toxicity and endocytosis of spinocerebellar ataxia type 6 polyglutamine domains: role of myosin IIb. Traffic.

[48] Getty AL, Benedict JW, Pearce DA (2011). A novel interaction of CLN3 with nonmuscle myosin-IIB and defects in cell motility of Cln3(−/−) cells. Exp Cell Res.

[49] Jensen CS, Watanabe S, Rasmussen HB, Schmitt N, Olesen SP, Frost NA, Blanpied TA, Misonou H (2014). Specific sorting and post-Golgi trafficking of dendritic potassium channels in living neurons. J Biol Chem.

[50] Correia SS, Bassani S, Brown TC, Lise MF, Backos DS, El-Husseini A, Passafaro M, Esteban JA (2008). Motor protein-dependent transport of AMPA receptors into spines during long-term potentiation. Nat Neurosci.

[51] Sun Y, Chiu TT, Foley KP, Bilan PJ, Klip A (2014). Myosin Va mediates Rab8A-regulated GLUT4 vesicle exocytosis in insulin-stimulated muscle cells. Mol Biol Cell.

[52] Langford GM (2002). Myosin-V, a versatile motor for short-range vesicle transport. Traffic.

[53] Oberhofer A, Spieler P, Rosenfeld Y, Stepp WL, Cleetus A, Hume AN, Mueller-Planitz F, Okten Z (2017). Myosin Va's adaptor protein melanophilin enforces track selection on the microtubule and actin networks in vitro. Proc Natl Acad Sci U S A.

[54] Varadi A, Tsuboi T, Rutter GA (2005). Myosin Va transports dense core secretory vesicles in pancreatic MIN6 beta-cells. Mol Biol Cell.

[55] Wada F, Nakata A, Tatsu Y, Ooashi N, Fukuda T, Nabetani T, Kamiguchi H (2016). Myosin Va and endoplasmic reticulum Calcium Channel complex regulates membrane export during axon guidance. Cell Rep.

[56] Wagner W, Brenowitz SD, Hammer JA (2011). Myosin-Va transports the endoplasmic reticulum into the dendritic spines of Purkinje neurons. Nat Cell Biol.

[57] He F, Wollscheid HP, Nowicka U, Biancospino M, Valentini E, Ehlinger A, Acconcia F, Magistrati E, Polo S, Walters KJ (2016). Myosin VI contains a compact structural motif that binds to ubiquitin chains. Cell Rep.

[58] Tumbarello DA, Kendrick-Jones J, Buss F (2013). Myosin VI and its cargo adaptors - linking endocytosis and autophagy. J Cell Sci.

[59] Sweeney HL, Houdusse A (2010). Myosin VI rewrites the rules for myosin motors. Cell.

[60] Spudich JA, Sivaramakrishnan S (2010). Myosin VI: an innovative motor that challenged the swinging lever arm hypothesis. Nat Rev Mol Cell Biol.

[61] Krapivinsky G, Krapivinsky L, Stotz SC, Manasian Y, Clapham DE (2011). POST, partner of stromal interaction molecule 1 (STIM1), targets STIM1 to multiple transporters. Proc Natl Acad Sci U S A.

[62] de Juan-Sanz J, Nunez E, Villarejo-Lopez L, Perez-Hernandez D, Rodriguez-Fraticelli AE, Lopez-Corcuera B, Vazquez J, Aragon C (2013). Na+/K+-ATPase is a new interacting partner for the neuronal glycine transporter GlyT2 that downregulates its expression in vitro and in vivo. J Neurosci.

[63] Shimizu H, Watanabe E, Hiyama TY, Nagakura A, Fujikawa A, Okado H, Yanagawa Y, Obata K, Noda M (2007). Glial Nax channels control lactate signaling to neurons for brain [Na+] sensing. Neuron.

[64] Berret E, Nehme B, Henry M, Toth K, Drolet G, Mouginot D (2013). Regulation of central Na+ detection requires the cooperative action of the NaX channel and alpha1 isoform of Na+/K+-ATPase in the Na+−sensor neuronal population. J Neurosci.

[65] Li KC, Zhang FX, Li CL, Wang F, Yu MY, Zhong YQ, Zhang KH, Lu YJ, Wang Q, Ma XL (2011). Follistatin-like 1 suppresses sensory afferent transmission by activating Na+,K+-ATPase. Neuron.

[66] Rose EM, Koo JC, Antflick JE, Ahmed SM, Angers S, Hampson DR (2009). Glutamate transporter coupling to Na,K-ATPase. J Neurosci.

[67] Illarionova NB, Gunnarson E, Li Y, Brismar H, Bondar A, Zelenin S, Aperia A (2010). Functional and molecular interactions between aquaporins and Na,K-ATPase. Neuroscience.

[68] Zhang D, Hou Q, Wang M, Lin A, Jarzylo L, Navis A, Raissi A, Liu F, Man HY (2009). Na,K-ATPase activity regulates AMPA receptor turnover through proteasome-mediated proteolysis. J Neurosci.

[69] Zatti A, Chauvet V, Rajendran V, Kimura T, Pagel P, Caplan MJ (2005). The C-terminal tail of the polycystin-1 protein interacts with the Na,K-ATPase alpha-subunit. Mol Biol Cell.

[70] Beggah AT, Geering K (1997). Alpha and beta subunits of Na,K-ATPase interact with BiP and calnexin. Ann N Y Acad Sci.

[71] Lee K, Jung J, Kim M, Guidotti G (2001). Interaction of the alpha subunit of Na,K-ATPase with cofilin. Biochem J.

[72] Ferrandi M, Salardi S, Tripodi G, Barassi P, Rivera R, Manunta P, Goldshleger R, Ferrari P, Bianchi G, Karlish SJ (1999). Evidence for an interaction between adducin and Na(+)-K(+)-ATPase: relation to genetic hypertension. Am J Phys.

[73] Devarajan P, Scaramuzzino DA, Morrow JS (1994). Ankyrin binds to two distinct cytoplasmic domains of Na,K-ATPase alpha subunit. Proc Natl Acad Sci U S A.

