# Supplementary material for: Multiple myosin motors interact with sodium/potassium-ATPase alpha 1 subunits
Source: Mol Brain. 2018 Aug 7;11:45. doi: 10.1186/s13041-018-0388-1 (PMC6081954; doi:10.1186/s13041-018-0388-1)
Supplement: Supplementary file 1 — Table S1. Antibodies used for immunoprecipitation (IP) assays. Table S2. Antibodies used for immunoblotting. Table S3. Various features of the cDNA constructs used in the study. (DOCX 21 kb) [file 13041_2018_388_MOESM1_ESM.docx]

Table S1. Antibodies used for immunoprecipitation (IP) assays*

| Antibodies | Company | Catalog no. | Clone | Isotype  (m=mouse,  r=rabbit, g=goat) | Antibodies (µg or µL) used per mL of tissue or cell lysate for IP |
| --- | --- | --- | --- | --- | --- |
| anti-Myh9 | Abcam | ab55456 |  | mIgG2b | 10 µg |
| anti-Myh10 | Abcam | ab684 | 3H2 | mIgG2b | 40 µL |
| anti-Myh14 | Abcam | ab191239 |  | rIgG | 10 µg |
| anti-MyoVa | Cell Signaling Technology | 3402S |  | rIgG | 40 µL |
| anti-MyoVI | Sigma-Aldrich | M0691 | MUD19 | mIgG1 | 20 µg |
| anti-Na/K-ATPase α-1 | DSHB | a6F |  | mIgG2a | 5 µg |
|  | EMD Millipore | 05-369 | C464.6 | mIgG1 | 5 µg |
| anti-KIF5B | EMD Millipore | MAB1614 | H2 | mIgG2b | 10 µg |
| anti-GFP | Thermo Fisher Scientific | A-11122 |  | rIgG | 5 µg |
|  | Abcam | ab1218 | 9F9.F9 | mIgG1 | 5 µg |
|  | Abcam | ab5450 |  | gIgG | 5 µg |
| anti-mCherry | Abcam | ab183628 |  | rIgG | 5 µg |
| mIgG1 [MOPC -21] | Abcam | ab18437 |  |  |  |
| mIgG2b [MG2b-57] | Abcam | ab18428 |  |  |  |
| rIgG | Thermo Fisher Scientific | 10500C |  |  |  |
| gIgG | Santa Cruz Biotechnology | sc-2028 |  |  |  |

* Whenever more than one antibody for the IP of the same antigen is used, the first one listed in the table is the default one and the other ones are specifically noted wherever they are used.

Table S2. Antibodies used for immunoblotting*

| Antigen  molecular  weight (kDa) | Antibodies | Company | Catalog no. | Clone | Isotype  (m=mouse,  r=rabbit) | Antibody dilutions/  concentrations used in IB assay |
| --- | --- | --- | --- | --- | --- | --- |
| 220-26 | anti-MYH9 | Abcam | ab55456 |  | mIgG2b | 1:500 |
| 200 | anti-MYH10 | Abcam | ab684 | 3H2 | mIgG2b | 1:1000 |
| 207/190 | anti-Myosin Va | Cell Signaling Technology | 3402S |  | rIgG | 1:500 |
| ~150 | anti-Myosin VI | Sigma | M0691 | MUD19 | mIgG1 | 1:500 |
| 260 | anti-pan-Na_v_α | Sigma-Aldrich | S8809 | K58/35 | mIgG1 | 1:1000 |
| 110 | anti-Na^+^/K^+^-ATPase α | Santa Cruz Biotechnology | sc-58628 | M7-PB-E9 | mIgG1 | 1:200 |
| 110 | anti-Na/K-ATPase α-1 | Abcam | ab7671 |  | mIgG1 | 1:500 |
|  |  | Millipore | 05-369 | C464.6 | mIgG1 | 1:1000 |
|  |  | DSHB | a6F |  | mIgG2a | 0.5 µg/mL |
| 42-45 | anti-beta-Actin | Sigma | A5441 | AC-15 | mIgG1 | 1:10,000 |
| 20 | anti-pan-MRCL | Santa Cruz Biotechnology | sc-28329 | E4 | mIgG1 | 1 µg/mL |
| 27-30 | anti-GFP | NeuroMab | 75-131 |  | mIgG2a | 1:1000 |
|  |  | Abcam | ab5450 |  | gIgG | 1:2000 |
| 27 | anti-mCherry | Abcam | ab183628 |  | rIgG | 1:1000 |

* Whenever more than one antibody for the detection of the same antigen is used, the first one listed in the table is the default one and the other ones are specifically noted wherever they are used.

Table S3. Various features of the cDNA constructs used in the study

| Clone/  construct | Species | Company (Catalog No.) | NCBI Accession No. | Isoform | AA  length | Actin binding site (ABS) | AA deletions for  Δ-tail constructs |
| --- | --- | --- | --- | --- | --- | --- | --- |
| Myh9 | Human | Addgene (11347) | NM_002473/  NP_002464 |  | 1960 | AAs: 654-676 | AAs: 1928-1960 |
| Myh10 | Human | Addgene (11348) | NM_005964/ NP_005955 | 2 | 1976 | AAs: 661-683 | AAs: 1934-1976 |
| Myh14 | Mouse | Addgene (10843) | NM_028021/  NP_082297 | 3 | 1992 | AAs: 674-696 | AAs: 1946-1992 |
| MyoVa | Human | Dharmacon  OHS5893-202503922 | NM_000259/  NP_000250 |  | 1855 |  |  |
|  |  |  |  |  |  |  |  |
| MyoVI | Human | Dharmacon  (EHS1001-213247774) | NM_004999/  NP_004990 | 1 | 1285 |  | ΔT1:1264-1285  ΔT2:1226-1285  ΔT3:1166-1285 |
| Na^+^/K^+^-ATPase α1 | Mouse | Dharmacon  (MMM1013-202769052) | NM_144900/  NP_659149 |  | 1023 |  |  |
| Ankyrin-G-270-mCherry | Rat | Addgene (42566) | NM_001033984/ NP_001029156 | 2 | 2622 |  |  |
