# Supplementary figures and images for: Multiple myosin motors interact with sodium/potassium-ATPase alpha 1 subunits
Source: Mol Brain. 2018 Aug 7;11:45. doi: 10.1186/s13041-018-0388-1 (PMC6081954; doi:10.1186/s13041-018-0388-1)

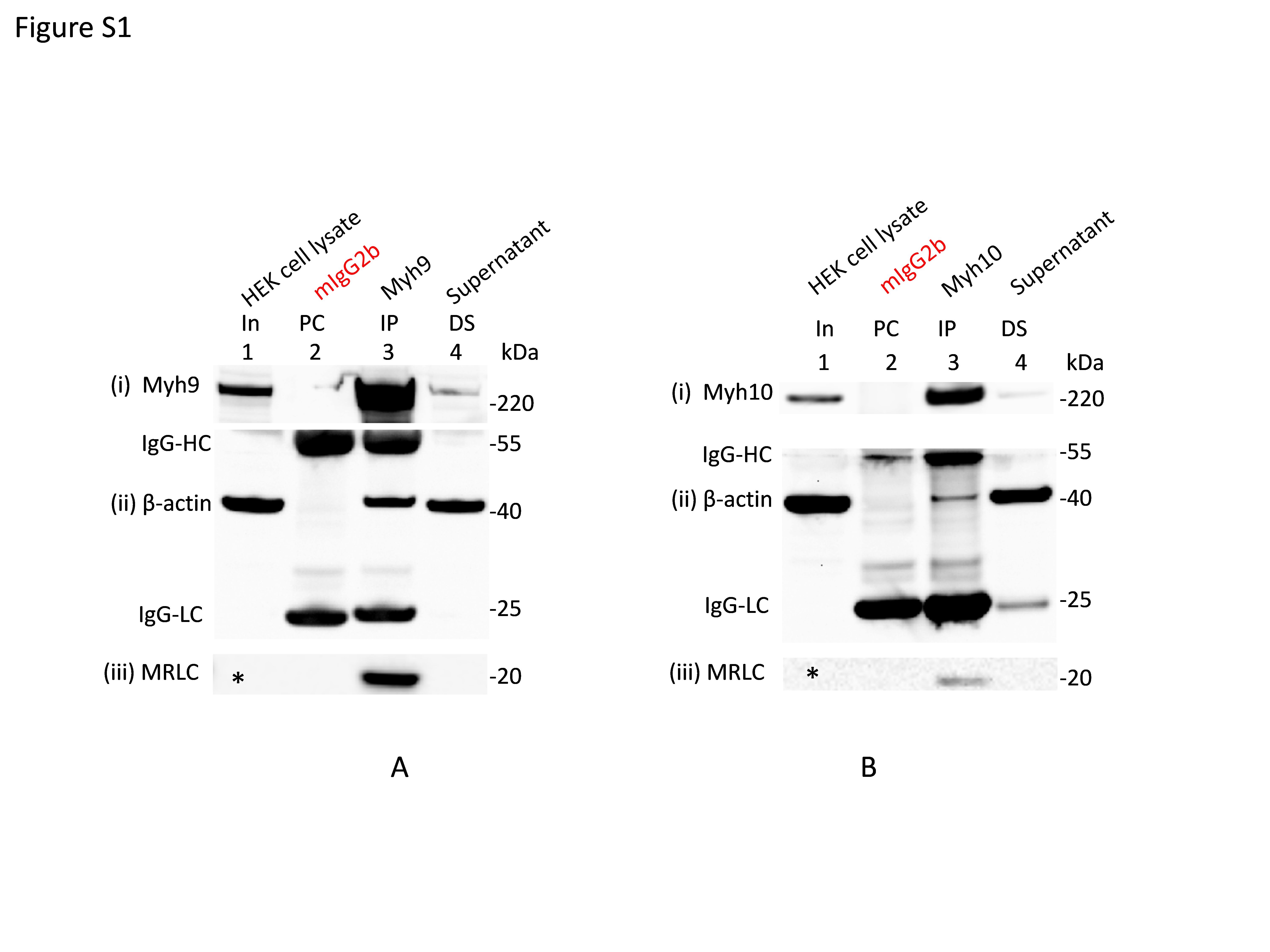

Supplement: Supplementary file 2 — Figure S1. Anti-myh9 and anti-myh10 antibodies immunoprecipitate their cognate antigens and/or co-immunoprecipitate partner proteins (such as β-actin and/or MRLCs) of their cognate antigens from HEK293 cells. Lysates of non-transfected HEK293 cells (In, lane 1 in A and B) were precleared (PC) with mouse IgG2b isotypes (mIgG2b; lane 2 in A and B) prior to immunoprecipitation (IP) using antibodies for myh9 (lane 3 in A) and myh10 (lane 3 B) of mIgG2b isotypes. IP complexes in the gel were loaded following the loading of their respective PC complexes. Myh9 immunoreactive bands in lane 3 of panel (i) in A and myh10 immunoreactive bands in lane 3 of panel (i) in B indicated immunoprecipitation of myh9 and myh10 by their respective antibodies. Presence of β-actin immunoreactive bands in the IP lanes of A (ii) and B (ii) indicated co-immunoprecipitation of it by myh9 and myh10 from non-transfected HEK293 cells. Both myh9 and myh10 also co-immunoprecipitated MRCLs (panel (iii) of A and B) from HEK293 cells. An asterisk (‘*’) in A and B indicates lack of detection of MRLCs in the input samples. Myh9 or myh10 immunoreactive bands in the depleted supernatant lanes (DS, lane 4 in panel (i) in A and B) indicate that both Mg2+-ATPases survive the IP procedure. Mouse IgG-HC and IgG-LC (panel (ii) in A and B) separated from their intact immunoglobulins (that is used for PC or IP) upon denaturation could be seen as this section of the blot is probed with mouse anti-β-actin antibodies. (TIF 1319 kb) [file 13041_2018_388_MOESM2_ESM.tif]

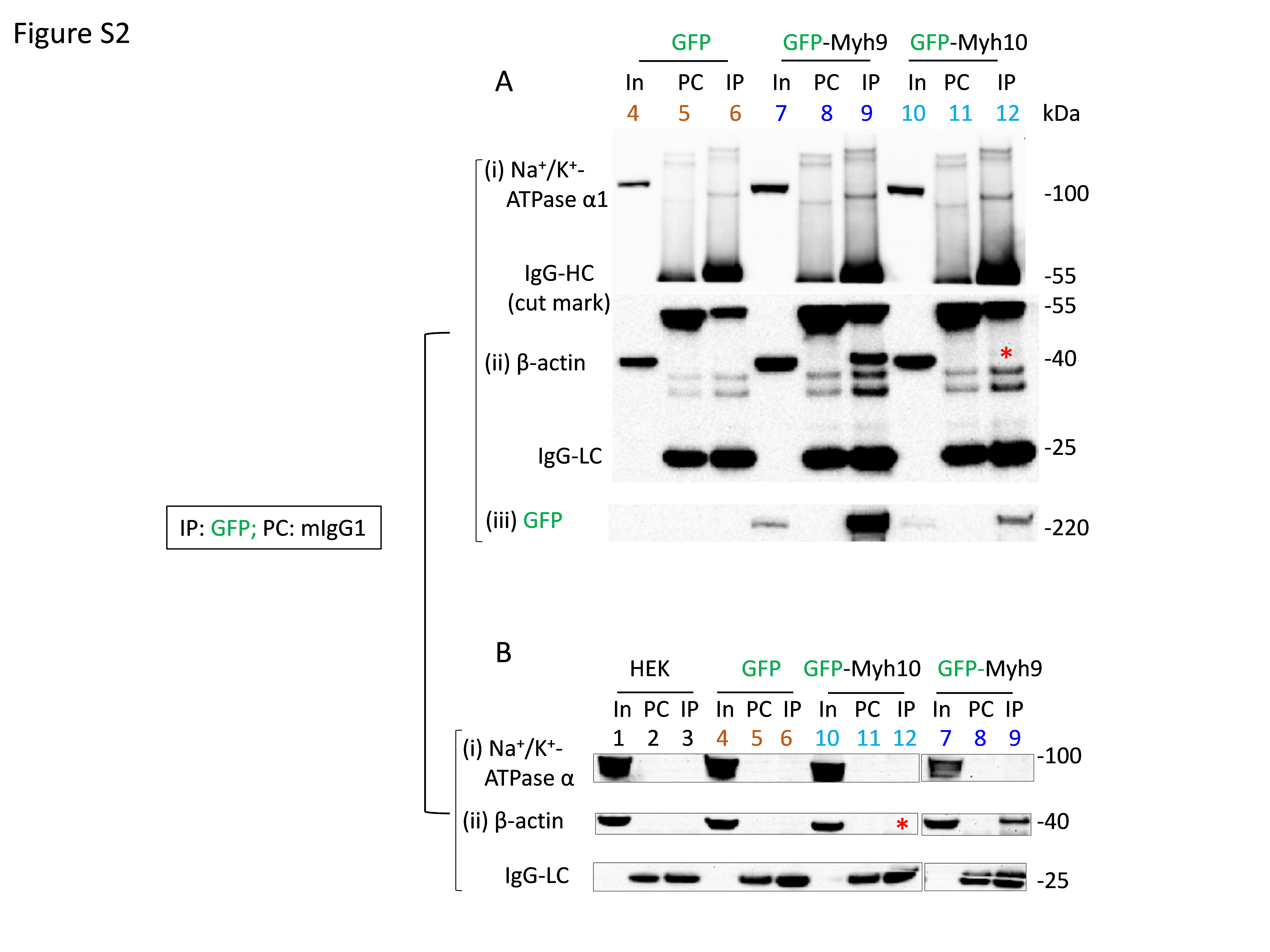

Supplement: Supplementary file 3 — Figure S2. Lack of co-immunoprecipitation of Na+/K+-ATPase α1 subunits by recombinant myh9 or myh10 tagged with GFP-in their N-termini. Lysates of non-transfected HEK293 cells (In; lane 1 in B) or HEK293 cells transiently transfected with GFP (In; lane 4 in A and B), GFP-myh9 (In; lane 7 in A and B) or GFP-myh10 (In; lane 10 in A and B) plasmids were precleared with mouse IgG1 isotypes (PC; lanes 2, 5, 8 and 11 in A or B) prior to immunoprecipitation using mouse anti-GFP antibodies (IP; lanes 3, 6, 9 and 12; Abcam: ab1218) of the IgG1 isotypes. Loading of PC complexes in the gel preceded those of the IP complexes. Na+/K+-ATPase α1 (Abcam: ab7671) immunoreactive bands in the input lanes 4, 7 and 10 but not in the PC or IP lanes 5, 6, 8, 9, 11 and 12 (A (i)) or Na+/K+-ATPase α (pan- Na+/K+-ATPase α) immunoreactive bands (Santa Cruz Biotechnology: sc-58,628) in the input lanes 1, 4, 7 and 10 but not in the PC or IP lanes 2, 3, 5, 6, 8, 9, 11 and 12 (B (ii)) indicated lack of co-immunoprecipitation of Na+/K+-ATPase α (or α1) subunits by N-terminally GFP tagged myh9 or myh10 expressed in HEK293 cells. GFP-myh9 (but not GFP-myh10) co-immunoprecipitated β-actin (lanes 9 vs. 12 in panel (ii) of A and B). Stripping and staining the uppermost section of the blot with rabbit anti-GFP antibodies indicated successful immunoprecipitation of GFP-myh9 (lane 9 in (iii) in A) and GFP-myh10 (lane 12 in (iii) in A) from HEK293 cell lysates. Denatured mouse IgG-HC and/or IgG-LC (iii) separated from their intact immunoglobulins (used in PC or IP reactions) are seen as the blot section is probed with mouse anti-β-actin antibodies. (TIF 2367 kb) [file 13041_2018_388_MOESM3_ESM.tif]

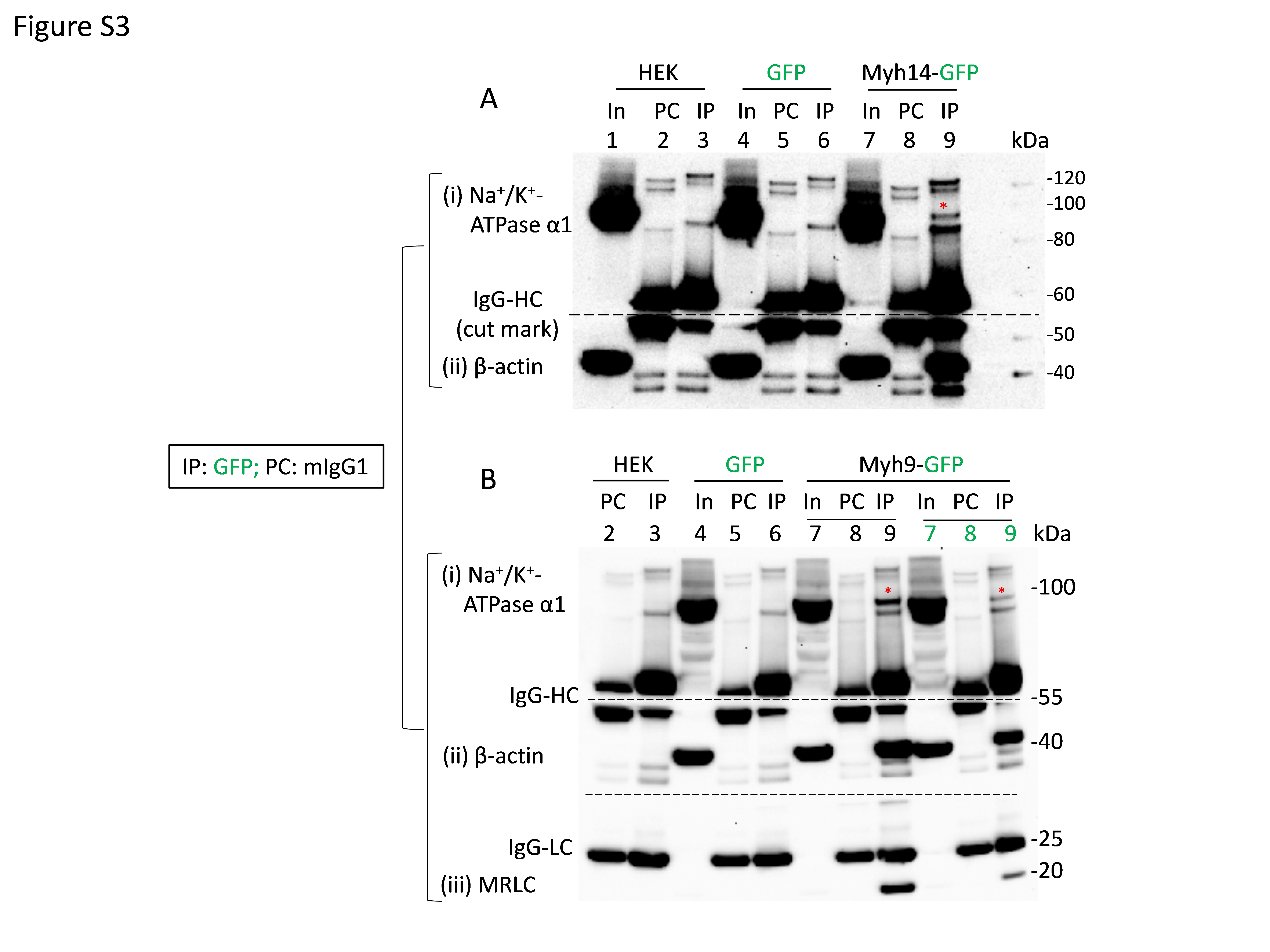

Supplement: Supplementary file 4 — Figure S3. Co-immunoprecipitation of Na+/K+-ATPase α1 subunits by C-terminally GFP tagged myh14 or myh9. Lysates of non-transfected HEK293 cells (In; lane 1 in A) or HEK293 cells transiently transfected with GFP (In; lane 4 in A and B), myh14-GFP (In; lane 7 in A) or myh9-GFP (In; lane 7 in B) plasmids were precleared with mouse IgG1 isotypes (PC; lanes 2, 5 and 8 in A and B) prior to immunoprecipitation using mouse anti-GFP antibodies (IP; lanes 3, 6 and 9 in A and B; Abcam: ab1218) of the IgG1 isotypes. Loading of PC complexes in the gel preceded those of the IP complexes. Na+/K+-ATPase α1 (Abcam: ab7671) immunoreactive bands in IP lane 9 (denoted by asterisk “*” in (i) in A and B) but not in any other IP or PC lanes indicated co-immunoprecipitation of Na+/K+-ATPase α1 subunits by C-terminally GFP tagged myh14 or myh9 expressed in HEK293 cells. Both myh14-GFP and myh9-GFP (but not GFP) co-immunoprecipitated β-actin (lane 9 in (ii) in A and B). Myh9-GFP (but not GFP) also co-immunoprecipitated MRLC (lane 9 in (iii) in B). Denatured mouse IgG-HC and/or IgG-LC separated from their intact immunoglobulins (used in PC or IP reactions) are observed as those blot sections are probed with mouse antibodies (for Na+/K+-ATPase α1, β-actin and/or MRLCs). Part of S3B is presented in Fig. 6a. (TIF 2846 kb) [file 13041_2018_388_MOESM4_ESM.tif]

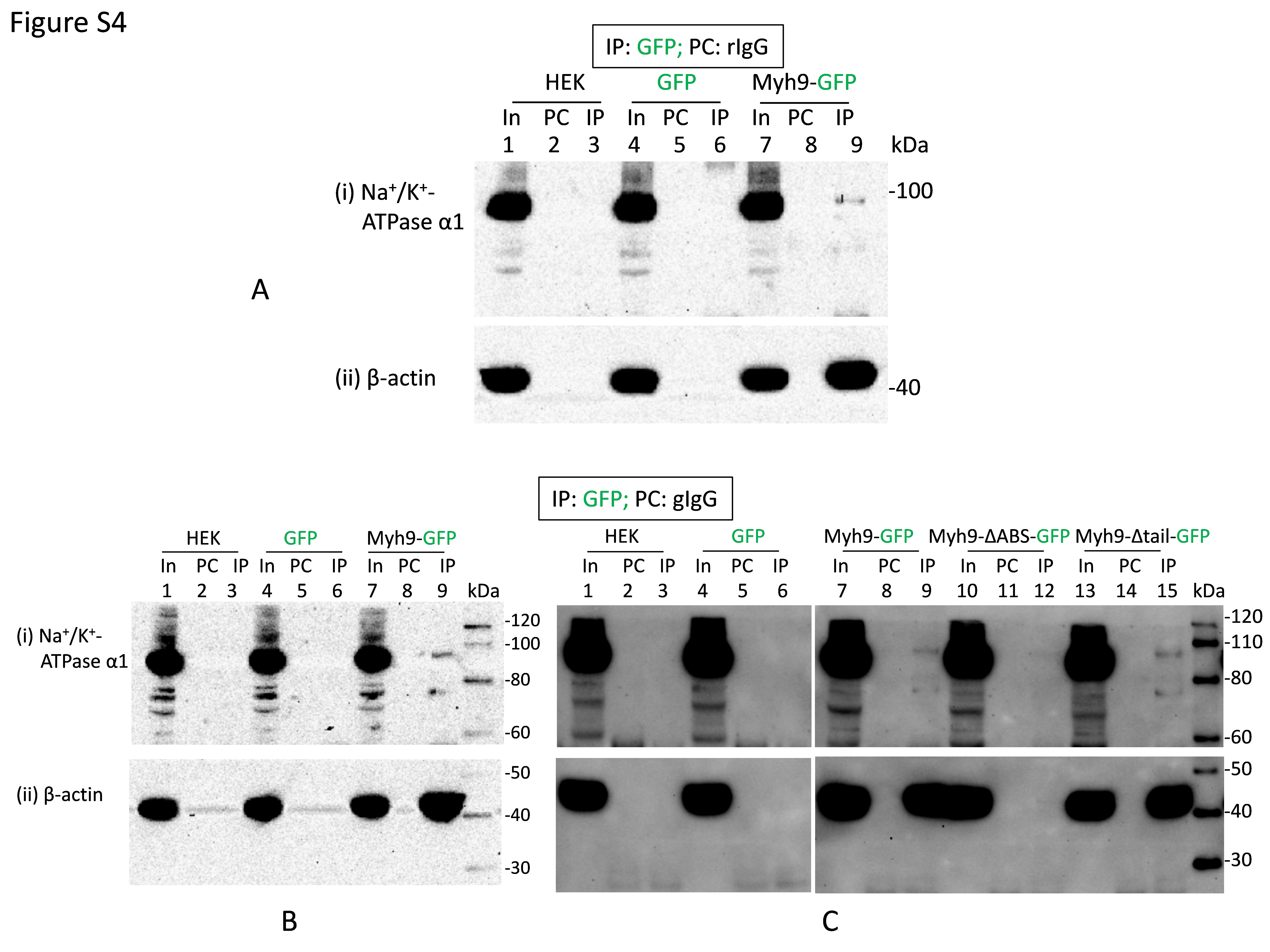

Supplement: Supplementary file 5 — Figure S4. Interaction of full length, actin binding site less (ΔABS) or tail-less (Δtail) recombinant myh9 with Na+/K+-ATPase α1 subunits and β-actin expressed in HEK293 cells. Lysates of non-transfected HEK293 cells (In; lane 1 in A, B and C) or HEK293 cells transiently transfected with GFP (In; lane 4 in A, B and C), myh9-GFP (In; lane 7 in A, B and C), myh9-ΔABS-GFP (In; lane 10 in C) or myh9-Δtail-GFP (In; lane 13 in C) plasmids (where the GFP tag is in their C-terminus) were precleared with rabbit IgG (PC; lanes 2, 5 and 8 in A) or goat IgG (PC; lanes 2, 5, 8, 11 and 14 in B or C) prior to immunoprecipitation using rabbit anti-GFP antibodies (IP; lanes 3, 6 and 9 in A) or goat anti-GFP antibodies (IP; lanes 3, 6, 9, 12 and 15 in B or C). Presence of obvious Na+/K+-ATPase α1 immunoreactive bands in lanes 1, 4, 7, 9, 10, 13 and 15 in A, B or C; greatly reduced Na+/K+-ATPase α1 immunoreactive bands in lane 12 in C; and absence of any Na+/K+-ATPase α1 immunoreactive bands in lanes 2, 3, 5, 6, 8, 11 and 14 in A, B or C indicated co-immunoprecipitation of Na+/K+-ATPase α1 subunits (panel (i)) from HEK293 cells transfected with myh9-GFP, myh9-ΔABS-GFP or myh9-Δtail-GFP plasmids thus confirming interaction between myh9 and Na+/K+-ATPase α1 subunits which is almost abrogated due to loss of actin binding site but not the tail regions in myh9. Myh9-GFP and myh9-Δtail-GFP, but not GFP, co-immunoprecipitated β-actin (lanes 9 and 15 respectively in panel (ii)). There was almost total loss of actin binding upon deletion of the actin binding site in myh9 (panel (ii), lane 12). Part of S4B is presented in Fig. 6b. (TIF 4636 kb) [file 13041_2018_388_MOESM5_ESM.tif]

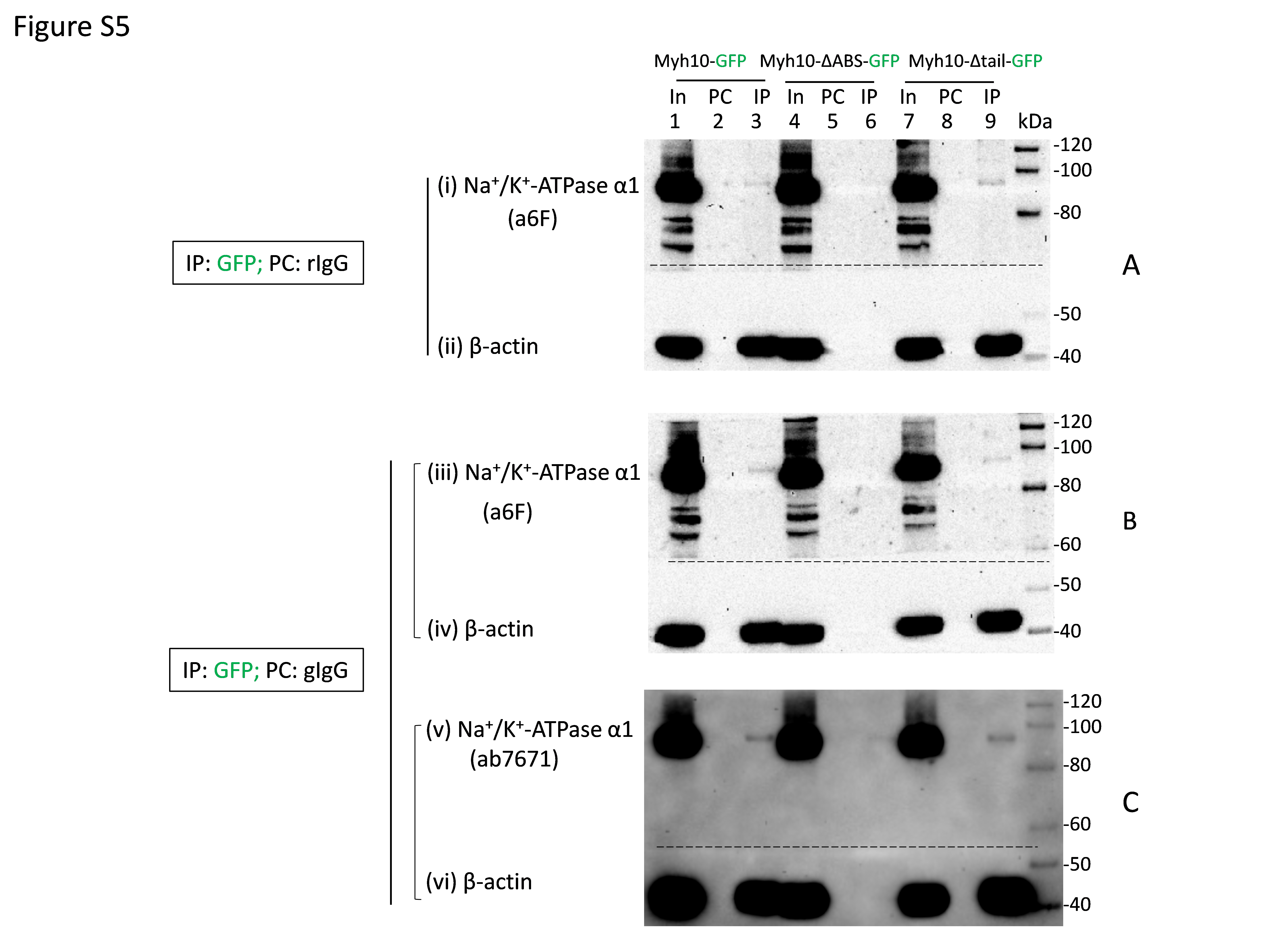

Supplement: Supplementary file 6 — Figure S5. Interaction of full length, actin-binding-site-less (ΔABS) or tail-less (Δtail) recombinant myh10 with Na+/K+-ATPase α1 subunits and β-actin expressed in HEK293 cells. Lysates of HEK293 cells transiently transfected with myh10-GFP (In; lane 1 in A, B and C), myh10-ΔABS-GFP (In; lane 4 in A, B and C) or myh10-Δtail-GFP (In; lane 7 in A, B and C) plasmids (where the GFP tag is in their C-terminus) were precleared with rabbit IgG (PC; lanes 2, 5 and 8 in A) or goat IgG (PC; lanes 2, 5 and 8 in B and C) prior to immunoprecipitation using rabbit anti-GFP antibodies (IP; lanes 3, 6 and 9 in A) or goat anti-GFP antibodies (IP; lanes 3, 6 and 9 in B and C). Loading of PC complexes in the gel preceded those of the IP complexes. Presence of Na+/K+-ATPase α1 immunoreactive bands in lanes 1, 3, 4, 7 and 9 (panel (i) in A, B and C) and greatly reduced or lack of presence of Na+/K+-ATPase α1 immunoreactive bands in lane 5 (panel (i) in A, B and C) indicated co-immunoprecipitation of Na+/K+-ATPase α1 subunits from HEK293 cells transfected with myh10-GFP or myh10-Δtail-GFP plasmids but not from those transfected with myh10-ΔABS-GFP plasmids thus confirming interaction between myh10 and Na+/K+-ATPase α1 subunits which is eliminated due to loss of actin binding site but not the tail regions in myh10. Myh10-GFP co-immunoprecipitated β-actin (lane 3 in (ii) in A, B and C) and there was complete loss of actin binding upon deletion of its actin binding site (lane 6 in (ii) in A, B and C). Tail-less myh10 also co-immunoprecipitated β-actin (lane 9 in (ii) in A, B and C). Control experiments for non-transfected HEK293 cells or HEK293 cells transiently transfected with GFP are done previously. (TIF 3566 kb) [file 13041_2018_388_MOESM6_ESM.tif]

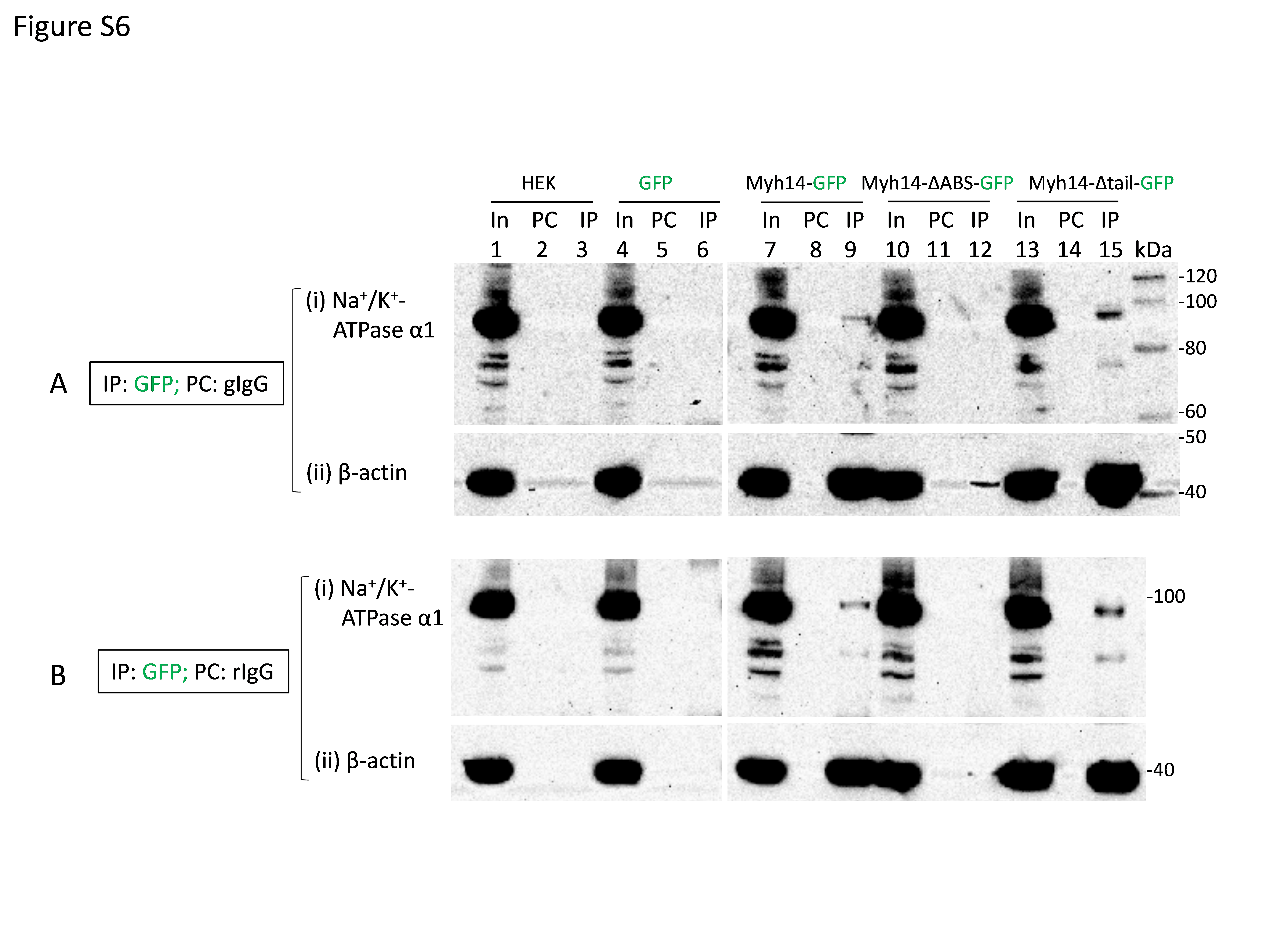

Supplement: Supplementary file 7 — Figure S6. Interaction of full length, actin binding site less (ΔABS) or tail-less (Δtail) recombinant myh14 with Na+/K+-ATPase α1 subunits and β-actin expressed in HEK293 cells. Lysates of non-transfected HEK293 cells (In; lane 1 in A and B) or HEK293 cells transiently transfected with GFP (In; lane 4 in A and B), myh14-GFP (In; lane 7 in A and B), myh14-ΔABS-GFP (In; lane 10 in A and B) or myh14-Δtail-GFP (In; lane 13 in A and B) plasmids (where the GFP tag is in their C-terminus) were precleared with rabbit IgG (PC; lanes 2, 5, 8 11 and 14 in A) or goat IgG (PC; lanes 2, 5, 8, 11 and 14 in B) prior to immunoprecipitation using rabbit anti-GFP antibodies (IP; lanes 3, 6, 9, 12 and 15 in A) or goat anti-GFP antibodies (IP; lanes 3, 6, 9, 12 and 15 in B). Presence of obvious Na+/K+-ATPase α1 immunoreactive bands in lanes 1, 4, 7, 9, 10, 13 and 15 in A or B; absence of Na+/K+-ATPase α1 immunoreactive bands in lane 12 in A or B; and absence of any Na+/K+-ATPase α1 immunoreactive bands in lanes 2, 3, 5, 6, 8, 11 and 14 in A or B indicated co-immunoprecipitation of Na+/K+-ATPase α1 subunits (panel (i)) from HEK293 cells transfected with myh14-GFP, myh14-ΔABS-GFP or myh14-Δtail-GFP plasmids thus confirming interaction between myh14 and Na+/K+-ATPase α1 subunits which is abrogated due to loss of actin binding site but not the tail regions in myh14. Myh14-GFP and myh14-Δtail-GFP co-immunoprecipitated β-actin (lanes 9 and 15 respectively in panel (ii) in A and B). There was almost total loss of actin binding upon deletion of the actin binding site in myh14 (panel (ii), lane 12 in A and B). Part of S6B is presented in Fig. 7d. (TIF 4560 kb) [file 13041_2018_388_MOESM7_ESM.tif]

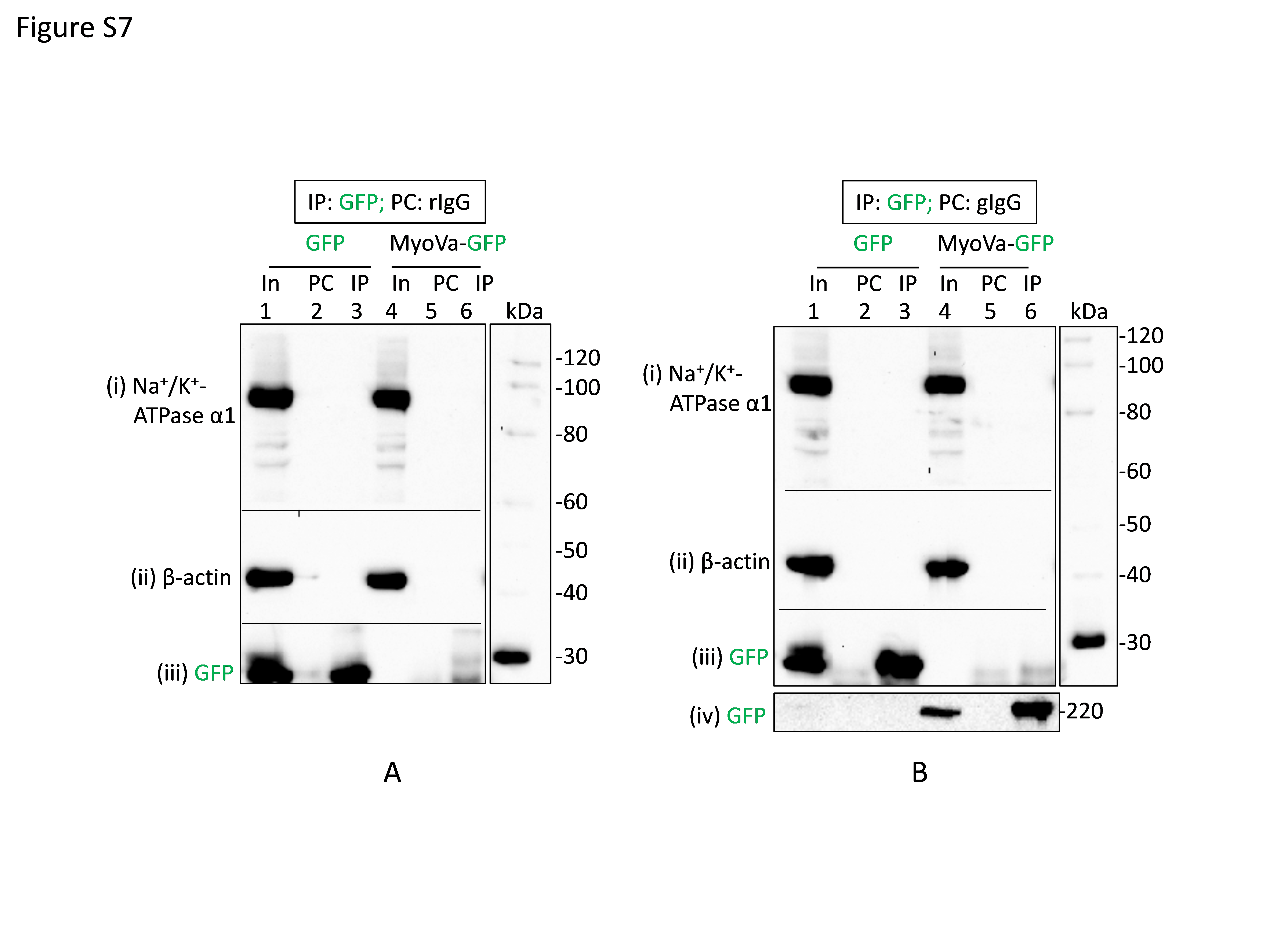

Supplement: Supplementary file 8 — Figure S7. Lack of co-immunoprecipitation of Na+/K+-ATPase α1 subunits and β-actin by recombinant myoVa. Lysates of HEK293 cells transiently transfected with GFP or myoVa-GFP plasmids were precleared with rabbit IgG (PC; lanes 2 and 5 in A) or goat IgG (PC; lanes 2 and 5 in B) prior to immunoprecipitation using rabbit anti-GFP antibodies (IP; lanes 3 and 6 in A) or goat rabbit anti-GFP antibodies (IP; lanes 3 and 6 in B). Loading of PC complexes in the gel preceded those of the IP complexes. Presence of Na+/K+-ATPase α1 immunoreactive bands in lanes 1 and 4, and absence of any Na+/K+-ATPase α1 immunoreactive bands in lanes 2, 3, 5 and 6 (panel (i) in A and B) indicated lack of co-immunoprecipitation of Na+/K+-ATPase α1 subunits from HEK293 cells transfected with myoVa-GFP. MyoVa-GFP did not co-immunoprecipitate β-actin (lane 6 in (ii) in A and B) from HEK293 cells. Staining the blots with mouse anti-GFP antibodies (NeuroMab: 75–131) indicated successful immunoprecipitation of GFP (lane 3 in (iii) in A and B) and myoVa-GFP (lane 6 in (iv) in B) from HEK293 cell lysates. (TIF 1906 kb) [file 13041_2018_388_MOESM8_ESM.tif]

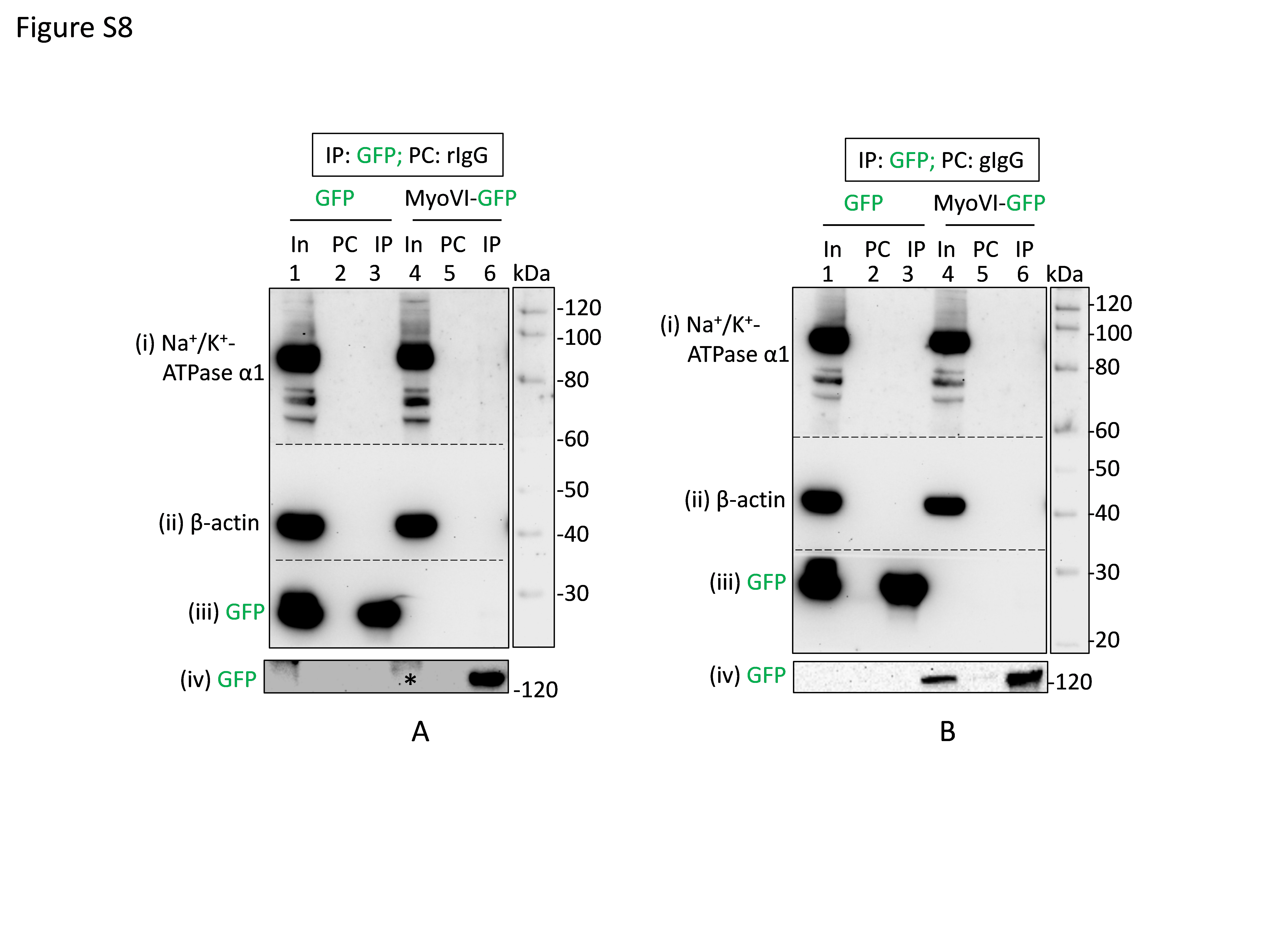

Supplement: Supplementary file 9 — Figure S8. Lack of co-immunoprecipitation of Na+/K+-ATPase α1 subunits and β-actin by recombinant myoVI. Lysates of HEK293 cells transiently transfected with GFP or myoVI-GFP plasmids were precleared with rabbit IgG (PC; lanes 2 and 5 in A) or goat IgG (PC; lanes 2 and 5 in B) prior to immunoprecipitation using rabbit anti-GFP antibodies (IP; lanes 3 and 6 in A) or goat anti-GFP antibodies (IP; lanes 3 and 6 in B). Loading of PC complexes in the gel preceded those of the IP complexes. Presence of Na+/K+-ATPase α1 immunoreactive bands in lanes 1 and 4, and absence of any Na+/K+-ATPase α1 immunoreactive bands in lanes 2, 3, 5 and 6 (panel (i) in A and B) indicated lack of co-immunoprecipitation of Na+/K+-ATPase α1 subunits from HEK293 cells transfected with myoVI-GFP. MyoVI-GFP did not co-immunoprecipitate β-actin (lane 6 in (ii) in A and B) from HEK293 cells. Staining the blots with mouse anti-GFP antibodies (NeuroMab: 75–131) indicated successful immunoprecipitation of GFP (lane 3 in (iii) in A and B) and myoVI-GFP (lane 6 in (iv) in A and B) from HEK293 cell lysates. The input signal for myoVI-GFP (indicated with an asterisk ‘*’) appears to be lost during stripping and/or staining with anti-GFP antibodies (lane 4 in (iv)). (TIF 2117 kb) [file 13041_2018_388_MOESM9_ESM.tif]

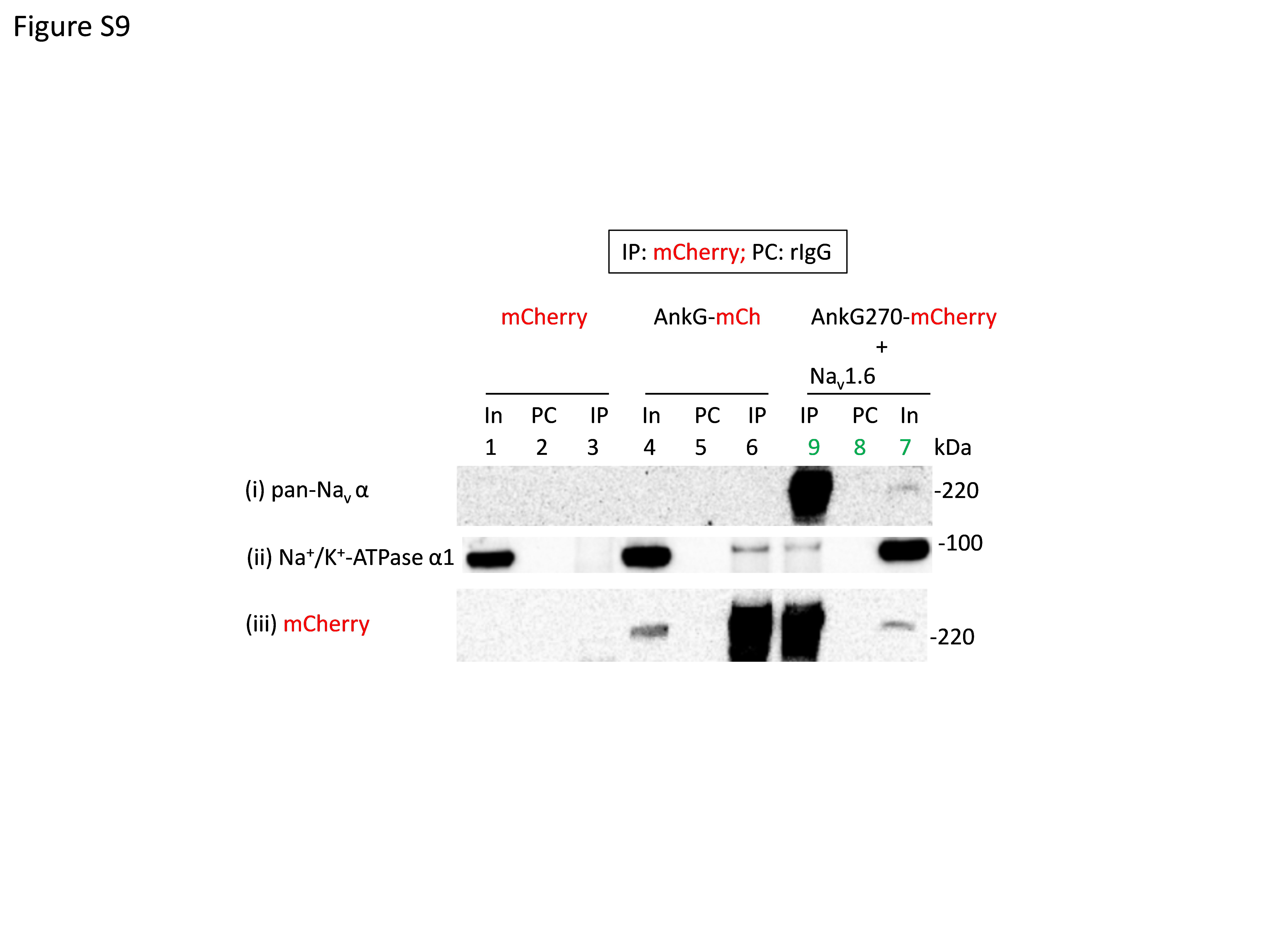

Supplement: Supplementary file 10 — Figure S9. mCherry antibodies are suitable for use in IP assay. Lysates of HEK293 cells transiently transfected with mCherry plasmids (In, lane 1) or ankyrin-G-mCherry plasmids (mCherry fused to the C-terminus: AnkG-mCh) (In, lane 4) or co-transfected with both AnkG-mCherry and Nav1.6 plasmids (In, lane 7) were precleared (PC) with rabbit IgG (PC; lanes 2, 5 and 8) prior to immunoprecipitation using rabbit anti-mCherry antibodies (IP; lanes 3, 6 and 9). Loading of PC complexes in the gel preceded those of the IP complexes. Pan-Navα immunoreactive bands (panel (i)) in IP lane 9 but not in other IP (i.e., 3 and 6) or PC lanes (2 and 5) indicated co-IP of Nav1.6 subunits (IP, lane 9) by recombinant ankyrin-G from HEK293 cells co-transfected with both ankyrin-G and Nav1.6 subunits but not from HEK293 cells transfected with mCherry or AnkG-mCherry. Similarly, Na+/K+-ATPase α1 immunoreactive bands (panel (ii)) in lanes 1, 4, 6, 7 and 9 (but not in lanes 2, 3, 5 and 8) indicated co-IP of Na+/K+-ATPase α1 subunits (i.e., lanes 6 and 9) by recombinant ankyrin-G from HEK293 cells transfected with recombinant AnkG alone or along with Nav1.6 subunits but not from HEK293 cells transfected with mCherry thus confirming interaction between AnkG and Na+/K+-ATPase α1 subunits. Also, mCherry immunoreactive bands in lanes 4, 6, 7 and 9 (panel (iii)) indicated IP of AnkG-mCherry by mCherry antibodies. (TIF 1055 kb) [file 13041_2018_388_MOESM10_ESM.tif]

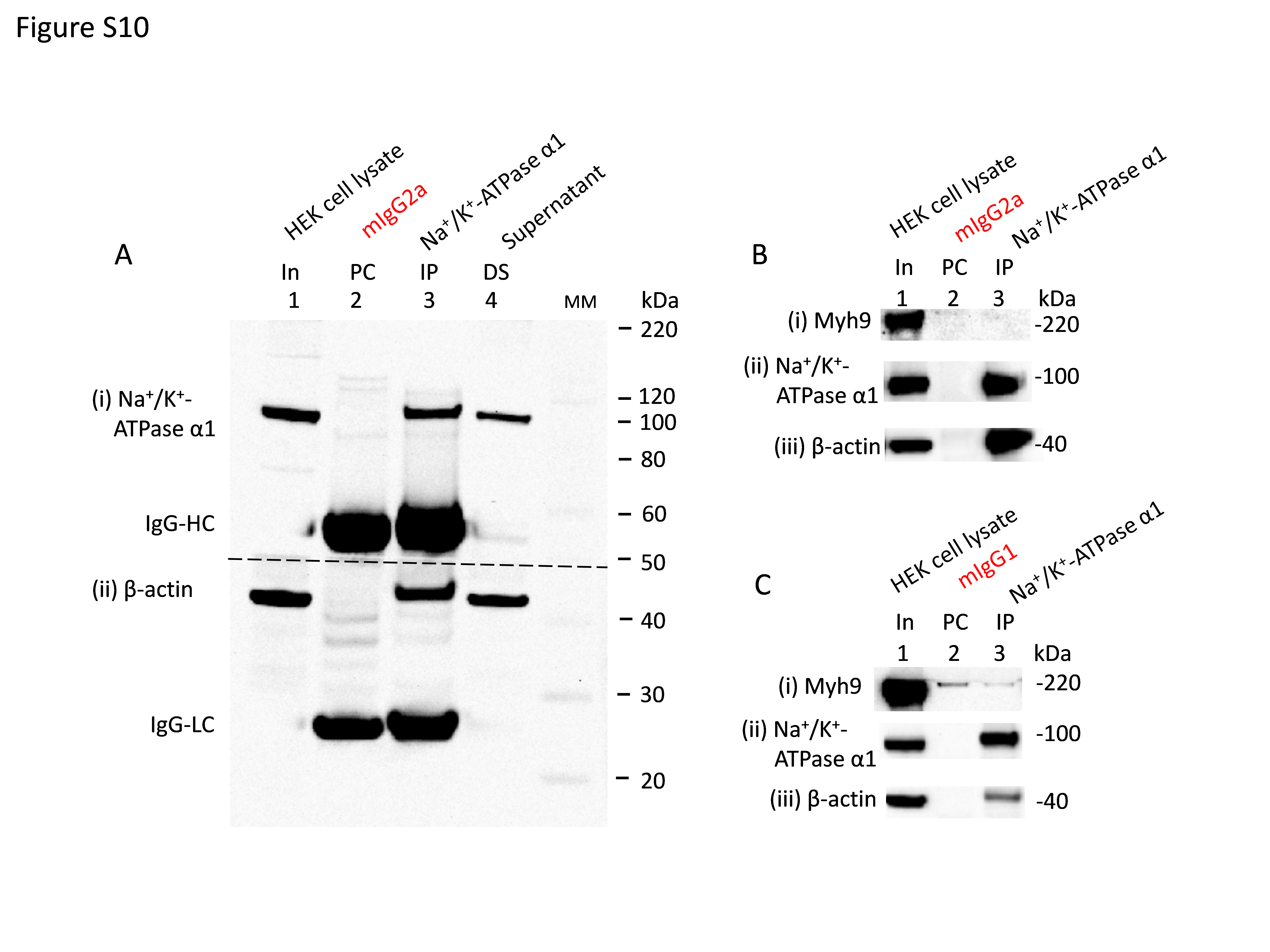

Supplement: Supplementary file 11 — Figure S10. Na+/K+-ATPase α1 subunits could not co-immunoprecipitate myh9 expressed in HEK293 cells. (A) Immunoprecipitation of Na+/K+-ATPase α1 subunits expressed in HEK293 cells. HEK293 cell lysates (In, lane 1) were precleared (PC, lane 2) with mouse IgG2a isotypes prior to immunoprecipitation (IP, lane 3) using mouse anti-Na+/K+-ATPase α1 antibodies (DSHB: a6F) of the IgG2a isotypes. Na+/K+ ATPase α1 immunoreactive bands in lanes 1, 3 and 5 but not in lane 2 (i) indicated immunoprecipitation of Na+/K+-ATPase α1 subunits expressed in HEK293 cells by the antibody in use. Na+/K+-ATPase α1 immunoreactive band was also observed in the depleted supernatant lane (DS, lane 4). As expected Na+/K+-ATPase α1 subunits also co-immunoprecipitated β-actin from HEK293 cells (iii). Denatured mouse IgG-HC (ii) and IgG-LC (iv) separated from their intact immunoglobulins (that is used for PC or IP) are seen as the blot was probed with mouse antibodies (for Na+/K+-ATPase α1 or β-actin). (B) and (C). Lack of co-immunoprecipitation of myh9 by Na+/K+-ATPase α1 subunits. HEK293 cell lysates (In, lane 1) were precleared (PC, lane 2) with indicated immunoglobulin isotypes (PC; mIgG2a in B and mIgG1 in C) prior to immunoprecipitation (IP, lane 3) using mouse anti-Na+/K+-ATPase α1 antibodies (DSHB: a6F in B and EMD Millipore; clone C464.6 in C). Antibodies for Na+/K+-ATPase α1 subunits could not co-immunoprecipitate myh9 (lane 3, panel (i) in B or C) though they could co-immunoprecipitate β-actin (lane 3, panel (iii) in B or C) and immunoprecipitate their cognate antigens (lane 3, panel (ii) in B or C) from HEK293 cells. (TIF 2570 kb) [file 13041_2018_388_MOESM11_ESM.tif]

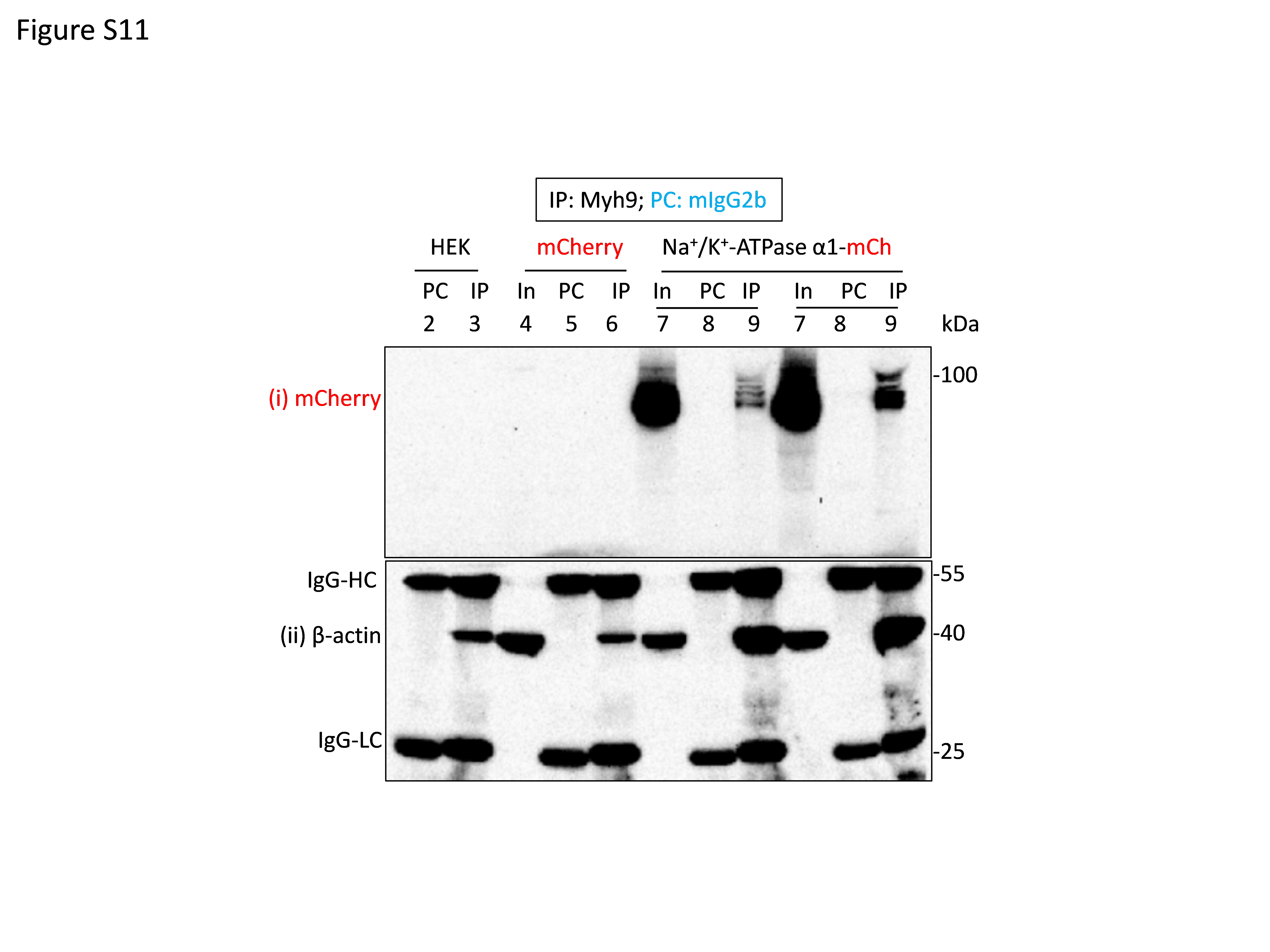

Supplement: Supplementary file 12 — Figure S11. Myh9 co-immunoprecipitate recombinant Na+/K+-ATPase α1 subunits expressed in HEK293 cells. Part of S11 is presented in Fig. 5a. (TIF 2388 kb) [file 13041_2018_388_MOESM12_ESM.tif]

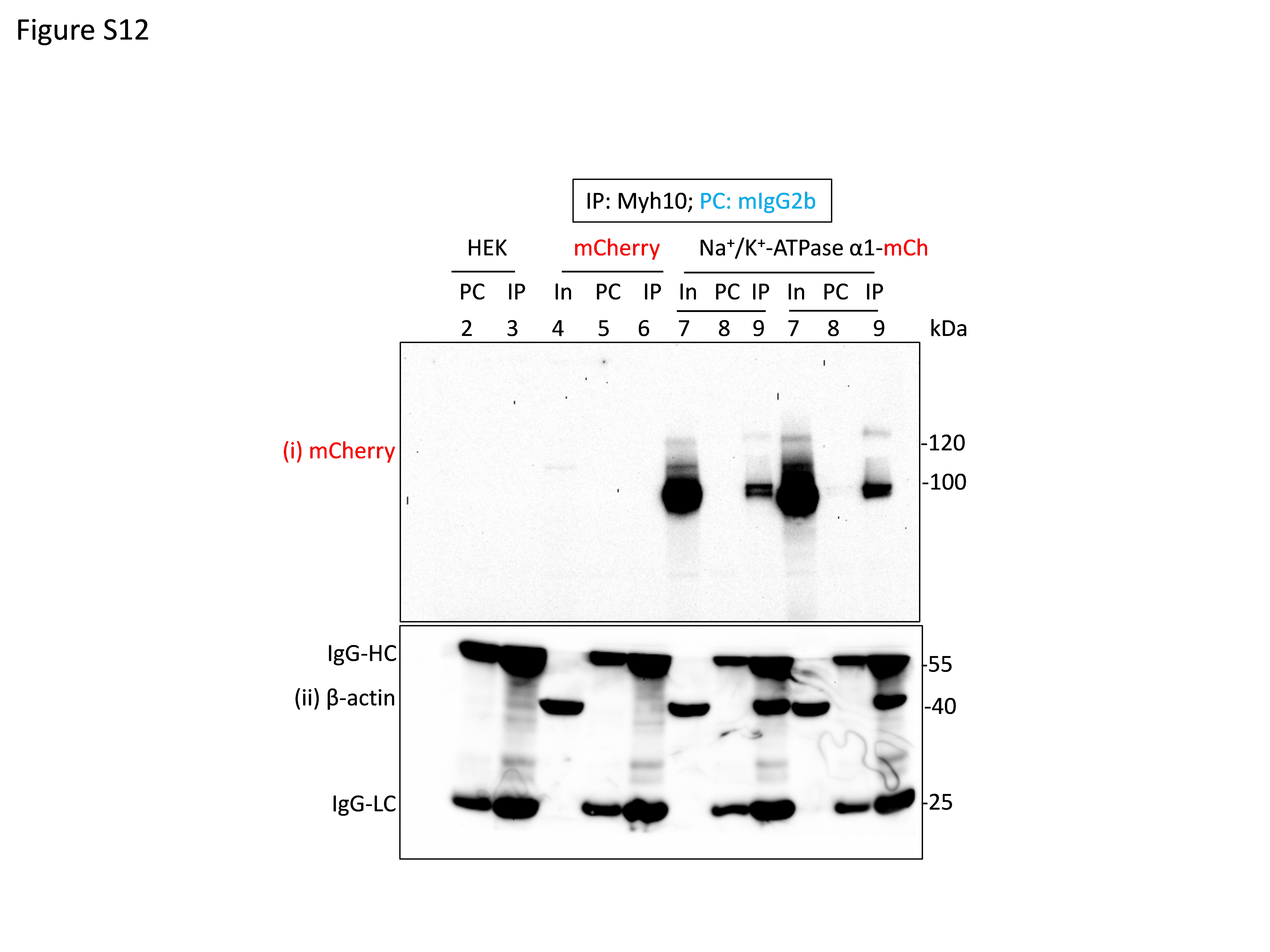

Supplement: Supplementary file 13 — Figure S12. Myh10 co-immunoprecipitate recombinant Na+/K+-ATPase α1 subunits expressed in HEK293 cells. Part of S12 is presented in Fig. 5b. (TIF 2619 kb) [file 13041_2018_388_MOESM13_ESM.tif]

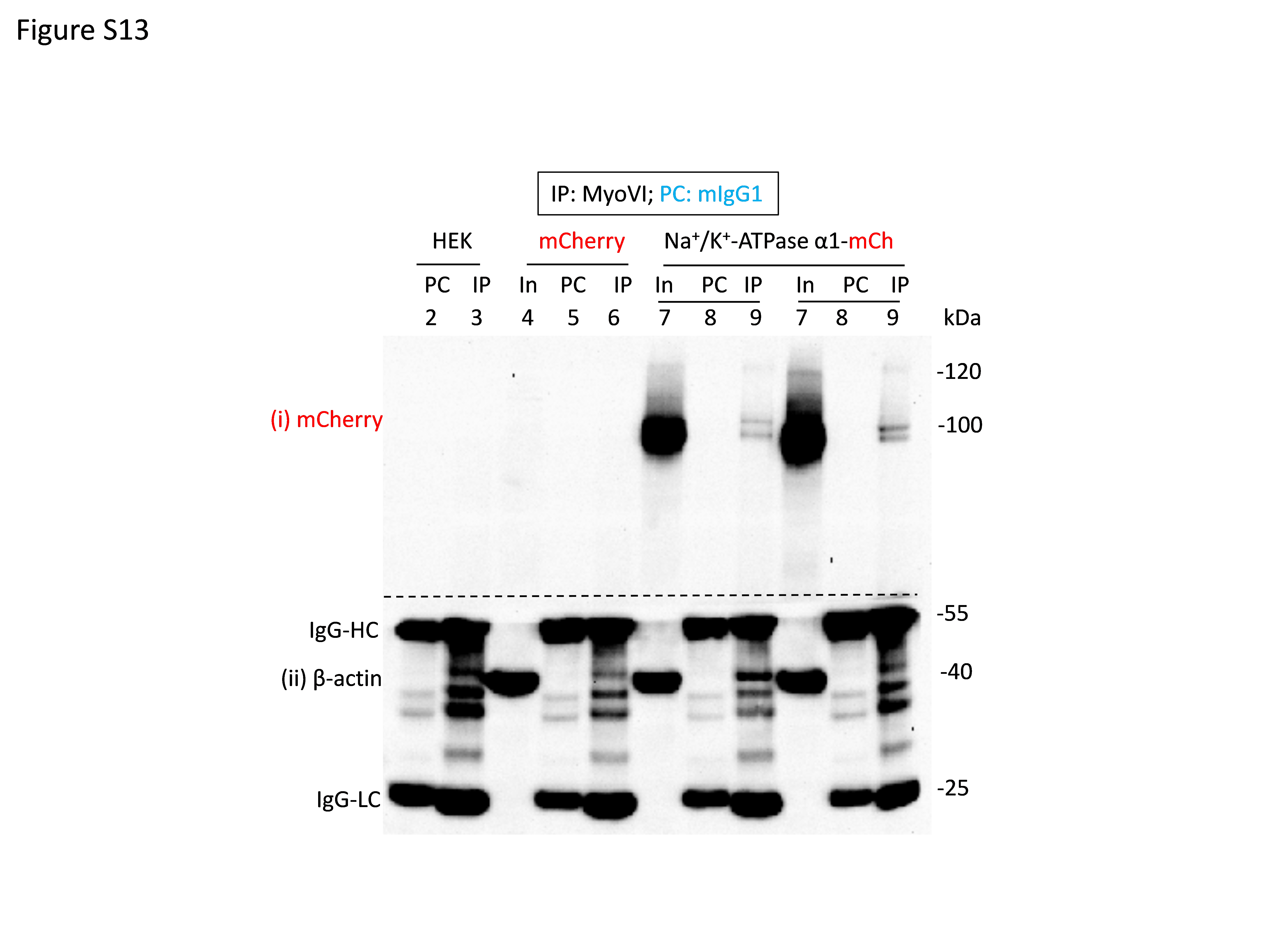

Supplement: Supplementary file 14 — Figure S13. MyoVI co-immunoprecipitate recombinant Na+/K+-ATPase α1 subunits expressed in HEK293 cells. Part of S13 is presented in Fig. 5c. (TIF 2302 kb) [file 13041_2018_388_MOESM14_ESM.tif]

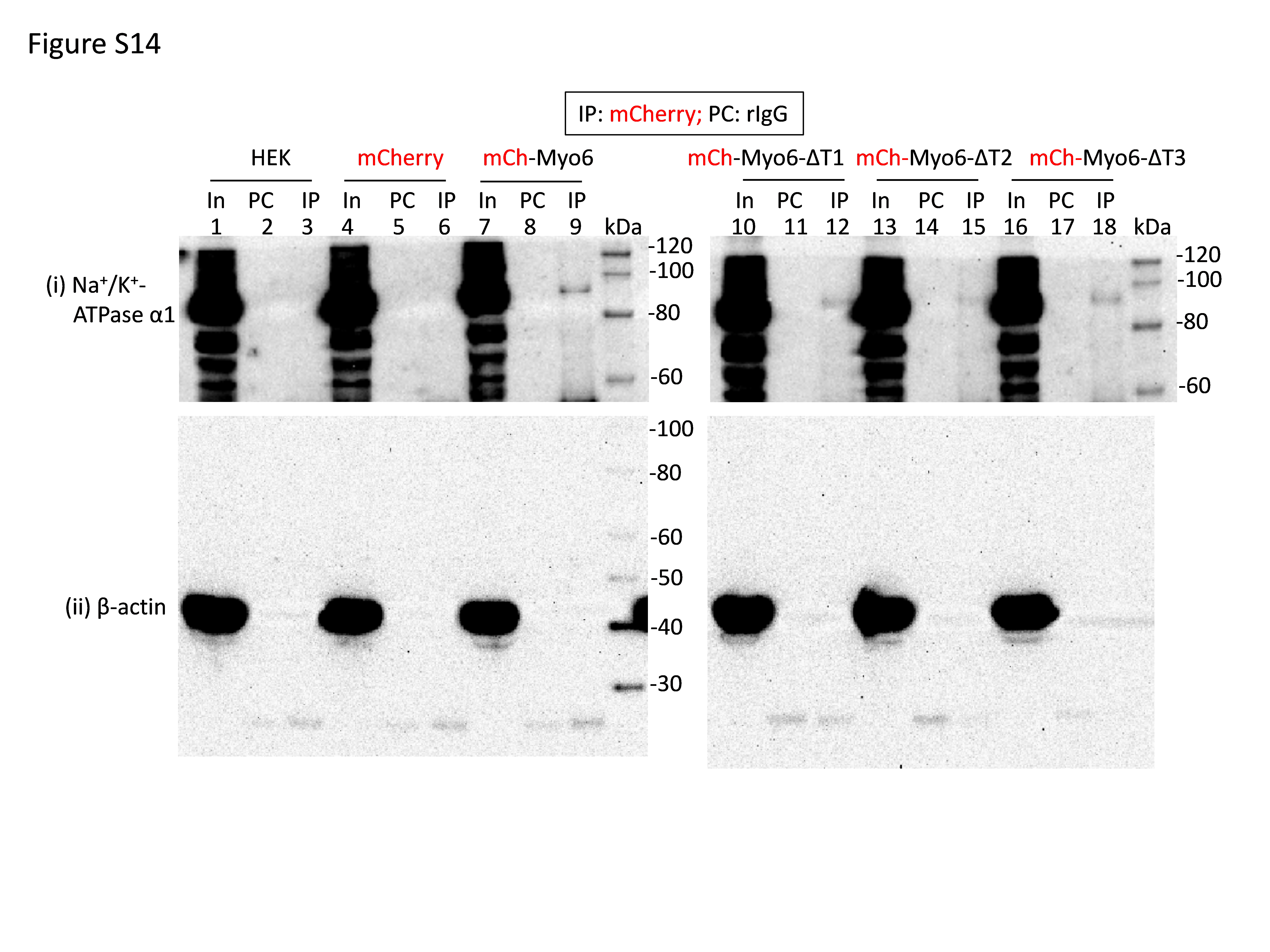

Supplement: Supplementary file 15 — Figure S14. Interaction of full length or tail-less (ΔT) recombinant myo6 with Na+/K+-ATPase α1 subunits expressed in HEK293 cells. Part of S14 is presented in Fig. 8c. (TIF 6409 kb) [file 13041_2018_388_MOESM15_ESM.tif]
